# A Review on Glial Fibrillary Acidic Protein Biosensors for Point-of-Care Stroke Diagnosis

**DOI:** 10.3390/s26206579

**Published:** 2026-10-16

**Authors:** Leonor C. Caetano, Vânia C. Pinto, Susana O. Catarino

**Affiliations:** 1 Center for Microelectromechanical Systems (CMEMS), University of Minho, 4800-058 Guimarães, Portugal; pg56139@alunos.uminho.pt (L.C.C.); vpinto@dei.uminho.pt (V.C.P.); 2 LABBELS—Associate Laboratory, 4800-122 Braga, Portugal

**Keywords:** biomarkers, biosensors, GFAP, PoC detection, stroke diagnosis

## Abstract

**Highlights:**

**What are the main findings?**

- Glial Fibrillary Acidic Protein (GFAP) is the most promising biomarker reviewed for hyperacute differentiation of intracerebral hemorrhage (ICH) from ischemic stroke (IS) and stroke mimics (SMs).
- Blood-based GFAP detection shows strong potential for rapid point-of-care (PoC) stroke diagnosis, although reported concentrations and diagnostic cutoffs vary across studies.

**What are the implications of the main findings?**

- Electrochemical, optical, mechanical, and emerging biosensor platforms could enable faster and more accessible GFAP testing in pre-hospital and PoC settings.
- Multiplexed biosensing approaches may improve stroke classification and patient stratification by combining GFAP with complementary biomarkers.

**Abstract:**

Stroke is a major cause of mortality and disability worldwide, and rapid differentiation between IS, haemorrhagic stroke (HS), and SMs is essential for timely treatment. This narrative review examines the potential of GFAP as a biomarker for acute stroke diagnosis and evaluates biosensing strategies for its detection, with particular emphasis on PoC and pre-hospital applications, profiting from its coupling with microfluidic technologies. The biological role, temporal kinetics, and diagnostic and prognostic value of GFAP are first discussed in comparison with other stroke biomarkers. The suitability of cerebrospinal fluid (CSF), serum, plasma, whole blood, urine, saliva, and sweat as biological matrices is then assessed. Finally, electrochemical, optical, mechanical, and other biosensor platforms developed for GFAP detection are reviewed in terms of sensing principles, analytical performance, biological matrices, assay duration, and potential clinical applicability. Overall, despite the heterogeneity of the data available in the literature, the reviewed evidence supports the potential utility of GFAP for hyperacute stroke assessment, owing to its rapid elevation following ICH and its ability to differentiate HS from IS and SMs. However, variability in sampling time, analytical methods, biological matrices, and patient populations limits direct comparison between studies. Further development of integrated and multiplexed PoC biosensors, also benefiting from the wide possibilities of advanced microfluidics’ technologies, may facilitate rapid and accessible stroke diagnosis and patient stratification.

## 1. Introduction

Stroke, or cerebrovascular accident (CVA), is an abrupt neurological event caused by impaired blood flow to the brain, leading to cell necrosis in affected brain regions [1,2]. The deficiency in blood supply typically arises either from a blockage that impedes blood circulation to the brain, referred to as an IS, or from the disruption of a cerebral blood vessel, causing a HS [2]. HS comprises two distinct forms: subarachnoid hemorrhage (SAH), which denotes haemorrhage in the subarachnoid space, and ICH, which entails bleeding occurring in the brain parenchyma [3].

A comprehensive evaluation study, published by the Global Burden of Disease, in 2024, assessed the occurrence, mortality, prevalence and disability-adjusted life years (DALYs) related to stroke across 204 countries between 1990 and 2021, and reaffirmed that stroke persists as a significant contributor to worldwide morbidity and mortality [4]. In 2021, it was estimated that approximately 11.9 million individuals experienced a stroke globally, with around 93.8 million people living as stroke survivors worldwide. Within the same year, approximately 7.3 million deaths occurred due to stroke, accounting for around 10.7% of global mortality, and 160.5 million DALYs, corresponding to 5.6% of total global DALYs [4]. Globally, IS accounted for about 65.3% of all stroke cases, ICH made up 28.8%, and SAH represented 5.8% of them [4]. During 2021, stroke ranked third among global causes of death, following COVID-19 and ischemic heart disease, and was fourth regarding DALYs, following COVID-19, ischemic heart disease, and neonatal disorders [4].

Looking at the Global Stroke Fact Sheet 2025, from the World Stroke Organization, which drew upon the findings from the previously referred study on stroke burden, there was a significant increase in stroke cases from 1990 to 2021 [4,5]. More specifically, incident strokes rose by 70.0%, deaths related to stroke increased by 44.0%, prevalent strokes grew by 86.0%, and DALYs increased by 32.0%. Most of the global stroke burden occurred in lower-income and lower-middle-income countries, being responsible for 89.0% of DALYs and 87.0% of stroke-related deaths [4,5]. Additionally, strokes linked to metabolic factors made up 69.0% of all cases, while those associated with environmental and behavioural risks accounted for 37.0% and 35.0% respectively [4,5]. Lastly, it was estimated that the global cost of stroke annually exceeds US$890 billion, representing 0.66% of the Gross Domestic Product (GDP) globally, with the projection of almost doubling until 2050 [5].

Considering this increase in stroke burden over the years, it is imperative to recognize and administrate effective prevention strategies to decrease the global impact of stroke. Prevention depends on the management of modifiable risk factors, which include hypertension, diabetes, dyslipidaemia, smoking and unhealthy lifestyle [6]. A meta-analysis and systematic review on strategies to control hypertension for secondary stroke prevention, published in 2025 [7], found that intensive control of blood pressure significantly reduced the risk of stroke recurrence, cardiovascular death, and myocardial infarctions, compared to the absence of treatment or the use of placebos [7]. A large-scale systematic review conducted by Wu et al. in 2024 [8], which included 71 prospective cohort studies, reported that individuals maintaining the healthiest combination of lifestyle behaviours, which included a balanced diet, not smoking, maintaining a healthy weight, regular physical activity, adequate sleep, and strong social engagement, had substantially lowered the risks of cardiovascular disease incidence, recurrence and mortality [8]. It was also found that, with a healthy lifestyle there was a reduction of 60.0% for the risk of stroke [8]. Regular leisure-time physical activity was proved especially beneficial, with a 2024 systematic review from Santis et al. revealing that even moderate levels were linked to a decrease in stroke risk [9]. Additionally, in 2025, a meta-analysis from Ungvari et al. found evidence that following a Mediterranean dietary pattern has been correlated with a significant reduction in stroke risk up to 12.0% [10].

Despite significant advances in prevention, not all strokes can be avoided. Risk factors considered non-modifiable, such as sex, ethnicity, age, and genetic predisposition, continue to play a substantial role in susceptibility to stroke [11]. Furthermore, women present additional sex-specific risk determinants, including pregnancy-induced hypertension and other pregnancy-associated complications, endometriosis, the use of hormonal contraceptives, premature ovarian failure, and early menopause [12]. According to research by the University of California, Los Angeles, two million brain cells are damaged every minute during a stroke [13]. Consequently, early recognition of stroke symptoms remains essential to minimize neurological damage and improve survival rates [14].

To enhance awareness, international stroke organizations promote the BE FAST or FAST acronym, which summarizes the key warning signs and reinforces the importance of urgent action [15]:

- B for balance: unexpected disturbance in coordination or balance;
- E for eyes: acute disturbance or visual loss;
- F for face: unilateral facial weakness or asymmetry;
- A for arms: weakness or sensory loss in the arm;
- S for speech: impaired speech clarity or comprehension;
- T for time: seek immediate assistance from emergency services.

Despite the advances in prevention and management strategies, the rapid diagnosis of stroke remains a significant clinical challenge. Currently, stroke diagnosis relies heavily on neuroimaging techniques, namely magnetic resonance imaging (MRI), and computed tomography (CT) to discriminate ischemic from haemorrhagic types and to exclude other potential causes that mimic stroke symptoms [16,17]. However, these procedures are time-consuming, strokes are not always accurately detected during initial imaging, and MRI scanners are not always available, leading to delayed treatment initiation and poorer patient outcomes [17,18,19,20]. As the efficacy of acute stroke interventions depends heavily on time, there is an urgent demand for faster, dependable, and more accessible diagnostic methods to improve clinical decision-making and reduce the time to treatment [21].

PoC testing based on blood biomarkers has recently emerged as a promising solution to this problem [22,23,24]. By enabling rapid diagnosis and patient stratification outside the hospital environment, such technologies could drastically reduce the time to treatment and improve clinical outcomes [23,25]. Thus, the various biomarkers and their applications in the context of stroke will be explored through this manuscript, along with their suitability for early stroke detection. Finally, different PoC platforms developed for the detection of an important stroke biomarker, GFAP, will be reviewed and discussed. To the best of the authors’ knowledge, this is the first comprehensive and critical review, fully covering the GFAP-existing biosensors for stroke (but not limited to this pathology) and discussing not only their technical requirements and potential for PoC integration, but also their adaptability to different fluid matrices, clinical applicability and stroke diagnostics compatibility.

### Stroke Subtypes

As previously mentioned, strokes are generally classified into two main subtypes according to the underlying cause of injury: IS, resulting from vascular occlusion, and HS, resulting from vascular rupture [2]. While both lead to neuronal death and functional impairment, their underlying pathophysiological cascades differ significantly, a divergence that is critical for the discovery of diagnostic biomarkers.

On one hand, IS occurs when blood flow to the brain is suddenly interrupted by a blockage in a cerebral artery, resulting in a decrease in oxygen and glucose in the impacted tissue [2,26]. This event initiates a sequence of interconnected molecular and cellular processes, commonly referred to as the IS cascade [2,26]. While neurons within the ischemic core undergo rapid and irreversible injury, surrounding tissue in the ischemic penumbra remains temporarily viable and is highly sensitive to secondary damage [2,26]. The initial reduction in energy availability leads to failure of dependent purine adenosine triphosphate (ATP) ion pumps, disrupting the ionic homeostasis and leading to the accumulation of intracellular calcium [26]. This disturbance promotes excessive liberation of the excitatory neurotransmitter glutamate and sustained activation of glutamate receptors, a process known as excitotoxicity [2,26]. Calcium overload and excitotoxic signalling impair mitochondrial function, further decreasing ATP production and augmenting the creation of reactive oxygen species, contributing to oxidative stress [2,26]. In parallel, cellular injury triggers inflammatory responses mediated by activated microglia and infiltrating immune cells, which trigger the secretion of pro-inflammatory cytokines and compromise the blood–brain barrier (BBB) [2,27]. These processes collectively activate regulated cell death pathways, particularly apoptosis, which contribute to progressive neuronal loss over hours to days following the initial ischemic event [2,26].

Overall, the IS cascade represents a dynamic and multifactorial process involving impaired energy metabolism, excitotoxic mechanisms, oxidative stress, inflammatory responses and ultimately cell death [2,26,27]. These mechanisms give rise to measurable molecular changes in blood and CSF, forming the biological basis for the identification of circulating biomarkers that can support early detection and diagnosis of IS [2,26,27].

On the other hand, HS occurs when a cerebral blood vessel ruptures, causing blood to leak inside the brain parenchyma or subarachnoid space, leading to rapid mechanical injury of neural tissue [3,28]. Primary injury arises immediately after vessel rupture, and the expanding hematoma causes an increase in intracranial pressure and compression of adjacent structures, contributing to reduced local cerebral perfusion and acute neuronal damage [3,28]. Following the initial bleeding event, secondary injury mechanisms are activated and play a critical role in extending neurological damage [26,28]. The breakdown of extravasated blood releases hemoglobin and its iron-containing degradation products into the brain environment, and the iron promotes the formation of reactive oxygen species, enhancing oxidative damage and leading to oxidative modifications of lipids and proteins and disruption of mitochondrial activity [26,28,29]. In addition to oxidative stress, the presence of blood components activates resident microglia and recruits peripheral immune cells, triggering a robust neuroinflammatory response that releases pro-inflammatory cytokines and chemokines [28]. These inflammatory mediators further disrupt the BBB, exacerbate cerebral edema, and enhance leukocyte infiltration, collectively promoting neuronal injury [26,28]. Several controlled forms of cell death, such as apoptosis and necroptosis, ferroptosis and pyroptosis, have been implicated in HS, and these mechanisms are interlinked with oxidative stress, iron toxicity and inflammatory signalling. The involvement of diverse cell death processes reflects the complex and evolving nature of neuronal injury after hemorrhage [26,28,29].

In summary, HS is characterized by a dynamic cascade in which mechanical injury, oxidative stress, inflammation, BBB disruption and regulated cell death interact to cause progressive brain damage [3,26,28,29]. These biological processes result in measurable molecular changes in blood and CSF, providing a mechanistic basis for identifying biomarkers that could support early diagnosis and prognostic assessment in HS [28,29].

## 2. Stroke Biomarkers

In the early hyperacute phase, defined as the first 6 h following stroke symptom onset, rapid distinction between true stroke and SMs, followed by accurate subtype identification, is essential [25,30,31]. The ideal biomarker must reliably distinguish the immediate mechanical rupture of the BBB that characterizes ICH from the delayed cytotoxic edema of ischemia [25,32]. These differences determine the kinetic profiles of biomarkers, distinguishing proteins released immediately after vessel rupture from molecules that appear later due to necrotic cell death or secondary responses [25]. In case IS is identified, the focus shifts to evaluating the risk–benefit profile of reperfusion therapy. Biomarkers linked to BBB integrity, inflammatory activation, or extracellular matrix (ECM) degradation may help predict the likelihood of haemorrhagic transformation following intravenous thrombolysis. Prognosis also shapes patient management, informing decisions about monitoring intensity, cardiac assessment, and long-term follow-up. Biomarkers reflecting systemic stress, neurogenic cardiac injury, or underlying comorbidities can support patient stratification according to expected outcomes [33,34].

This section critically examines the main biomarker classes relevant to these aims, with a particular focus on their temporal kinetics, mechanistic plausibility, and reported diagnostic performance.

### 2.1. Biomarkers of Structural and Neural Injury

Biomarkers of structural and neural injury represent the primary category of stroke molecular indicators. These reflect damage to the cellular architecture of the brain, specifically glial cells and neurons, and include GFAP, Neurofilament Light Chain (NfL), Neuron-specific Enolase (NSE) and S100 Calcium-Binding Protein B (S100B).

#### 2.1.1. GFAP

GFAP, an intermediate filament protein primarily found in astrocytes, is widely recognized as a key indicator for quickly distinguishing stroke subtypes, owing to its high specificity for ICH [32]. Different primary clinical studies have reported before on this role [35,36]. Particularly, recent multicenter studies have demonstrated that GFAP levels are significantly increased in IS patients in comparison to SMs, enabling accurate distinction when diagnostic models are augmented with clinical severity scores, like the National Institutes of Health Stroke Scale (NIHSS) [30]. Furthermore, GFAP exhibits superior discriminative capacity in differentiating ICH from IS compared to other protein candidates, achieving high specificity and sensitivity in the early hyperacute phase [25,32]. This diagnostic capability is intrinsically linked to the temporal kinetics of the protein; GFAP levels peak within the first 2 to 6 h following haemorrhagic events, but can be detected within minutes, due to the immediate mechanical disruption of the BBB and astrocyte destruction [37]. Conversely, in IS, the liberation of GFAP into the bloodstream is typically delayed, reflecting a slower process of necrotic cell death and secondary BBB breakdown [38]. Compared with ICH, GFAP concentrations in IS remain lower during the early hyperacute phase, gradually increasing over the subsequent days and reaching peak concentrations approximately 48 to 72 h after stroke onset [39,40]. This distinct kinetic profile renders GFAP an ideal candidate for pre-hospital PoC, enabling the early exclusion of hemorrhage to facilitate faster triage for reperfusion therapies [41]. Beyond its diagnostic utility, GFAP serves as a robust prognostic indicator, where elevated serum levels correlate with larger infarct volumes, increased stroke severity, and worse functional outcomes at discharge [42]. Crucially, GFAP levels measured within the first 24 h offer predictive value regarding the safety of reperfusion therapy, as higher concentrations are indicative of an elevated risk for haemorrhagic transformation and periprocedural injury following mechanical thrombectomy or thrombolysis [42,43]. Thus, GFAP represents a multifaceted biomarker that not only aids in the immediate stratification of stroke patients, but also informs long-term prognosis and therapeutic safety profiles [38].

#### 2.1.2. NfL

NfL is a neuron-specific cytoskeletal protein which has garnered significant attention as a robust biomarker for quantifying neuroaxonal damage in stroke [37,38,44]. However, the temporal kinetics of NfL release present a significant challenge for its application as an early hyperacute pre-hospital biomarker, where serum levels typically begin to rise only days after symptom onset, peaking between 14 and 21 days, or even later in the subacute phase [37,38,44].

#### 2.1.3. NSE

NSE, a glycolytic isoenzyme localized primarily within neurons and neuroendocrine cells, has been extensively investigated as a marker for neuronal structural damage [38]. In terms of temporal kinetics, NSE typically rises within 3 to 12 h following symptom onset; consequently, it is not suitable as a diagnostic for immediate reperfusion decisions if used in isolation, as levels may remain below diagnostic thresholds during the earliest pre-hospital window [37,38].

#### 2.1.4. S100B

S100B, which is largely present in astrocytes, functions as a calcium-binding protein and is a well-established biomarker for central nervous system injury [25,38]. S100B offers significant prognostic value; specifically, persistent elevations in serum S100B have been identified as robust predictors of malignant infarction, cerebral edema, and haemorrhagic transformation [38,41]. However, S100B typically only peaks at 2 to 3 days, rising within 3 to 12 h, following ischemic onset, making it less suitable as a standalone diagnostic for immediate triage decisions [38,41].

### 2.2. Biomarkers of BBB Stability and Matrix Degradation

Closely linked to structural damage is the integrity of the BBB, a critical determinant of stroke outcome and therapeutic safety. The biomarkers characterizing BBB stability and matrix degradation include Matrix Metalloproteinase-9 (MMP-9), Occludin, Claudin-5 and Zonula Occludens-1 (ZO-1).

#### 2.2.1. MMP-9

MMP-9 is a zinc-dependent endopeptidase that contributes to ECM breakdown and compromises the BBB [25,44]. In the context of acute IS, elevated MMP-9 has a strong link to reperfusion injury, neutrophil infiltration, and the subsequent risk of haemorrhagic transformation following intravenous thrombolysis [25,41]. However, MMP-9 lacks sufficient discriminatory strength for IS versus HS and stroke diagnosis [25]. In early diagnosis, MMP-9 possesses significant prognostic value, as early elevations are strongly correlated with baseline stroke severity and final infarct volume, whilst serving as a robust independent predictor of haemorrhagic transformation and cerebral edema, thereby providing critical safety data for guiding reperfusion therapies [25,44,45,46].

#### 2.2.2. Occludin, Claudin-5 and ZO-1

Occludin, Claudin-5 and ZO-1 are essential tight junction proteins responsible for the structural stability of the BBB [25,30]. These biomarkers have recently emerged as high-precision biomarkers for the hyperacute identification of acute IS [30]. Regarding diagnostic capability, circulating levels of Claudin-5 and Occludin have been shown to be pronouncedly higher in acute IS patients in comparison to SMs, with Claudin-5 demonstrating a marked ability to reflect immediate vascular permeability changes that distinguish true ischemic events from functional mimics [30]. Similarly, ZO-1 levels are elevated in acute IS patients, providing a specific indicator of BBB structural disarrangement that aids in the early differentiation of stroke subtypes, although its sensitivity as a standalone marker can be variable [25,30]. In terms of temporal kinetics, these proteins are particularly valuable for pre-hospital detection. Claudin-5 and Occludin are released rapidly following the initial mechanical and ischemic disruption of the neurovascular unit, with elevated concentrations detectable within the first 12 h of onset, offering a potential window for hyperacute triage [30,45]. However, in the context of reperfusion therapy assessment and the prediction of haemorrhagic transformation, MMP-9 remains the superior and more extensively validated biomarker, as it is a direct mediator of the matrix degradation that leads to BBB failure, rather than solely a byproduct of its structural collapse [25].

### 2.3. Biomarkers for Inflammation and Oxidative Stress

Beyond structural and vascular damage, inflammation and oxidative stress are central to the ischemic cascade and secondary brain injury. The principal mediators utilized to monitor this response include Interleukins (ILs), Tumour Necrosis Factor α (TNF-α) and C-Reactive Protein (CRP).

#### 2.3.1. ILs

ILs, specifically Interleukin-6 (IL-6), are critical neuroinflammatory mediators in acute brain injury [25,37]. While IL-6 offers good sensitivity for stroke diagnosis and rapid temporal kinetics, potentially doubling within 26 min of onset, its use as a standalone pre-hospital marker is limited by low specificity [25,37,41]. With the distinction between HS and IS, IL-6 shows high specificity but a low sensitivity [25]. ILs only hold significant prognostic value within the first 24 to 72 h, not being suitable for pre-hospital evaluation [25,38,44].

#### 2.3.2. TNF-α

TNF-α, a pro-inflammatory cytokine liberated by perivascular microglia and macrophages within 4 h of cerebral ischemia, represents a critical component of the early neuroinflammatory cascade [37,44]. When analyzing diagnostic precision, TNF-α exhibits moderate specificity but low sensitivity in stroke prediction models, suggesting it is insufficient for distinguishing stroke from SMs or differentiating IS from HS [25]. From a prognostic perspective, elevated circulating TNF-α levels are robustly related to larger infarct volumes, heightened neuroinflammation, and an increased susceptibility to post-stroke infections, thereby serving as a reliable predictor of delayed recovery and adverse functional outcomes [38,41].

#### 2.3.3. CRP

CRP, which reflects systemic inflammatory activity, serves as a robust prognostic indicator in acute IS [37]. In the context of temporal kinetics, CRP has value in the subacute phase, meaning it is not suitable for pre-hospital application [38].

### 2.4. Biomarkers of Coagulation and Fibrinolysis

Distinct from neural injury, biomarkers of coagulation and fibrinolysis provide insight into the thrombotic activity underlying the ischemic event. One of these biomarkers is D-dimer, a product of fibrin breakdown, which has been explored as a marker of thrombotic activity in acute stroke [37,44]. Its effectiveness as an independent diagnostic marker for differentiating stroke from SMs is limited due to low specificity, as elevated levels can also occur in infections, malignant, trauma, thromboembolic, and other non-stroke disorders [37,38]. Moreover, D-dimer does not reliably distinguish IS from HS, showing a low sensitivity [25]. Furthermore, D-dimer levels serve as a predictor for safety in reperfusion therapies, and markedly high levels are associated with a heavy clot burden and an increased risk of haemorrhagic transformation post-thrombolysis, as well as a higher probability of recurrent ischemic events associated with occult atrial fibrillation or cancer [37,38]. Regarding temporal kinetics, D-dimer levels exhibit an early elevation within minutes, and peaks from 3 to 24 h of symptom onset, being a suitable biomarker for the pre-hospital care [38].

### 2.5. Biomarkers of Cardiac and Systemic Stress

Finally, given the intricate heart and brain connection, biomarkers of cardiac and systemic stress are also indispensable. These include cardiac Troponin I (TnI), B-Type Natriuretic Peptide (BNP) and N-Terminal Prohormone (NT-proBNP).

#### 2.5.1. TnI

Although TnI is traditionally viewed through the prism of myocardial injury, its utility in the acute stroke setting is profound, serving as a critical exclusionary tool for SMs such as acute myocardial infarction [25,47]. TnI concentrations elevate rapidly following the onset of stress, and this rise reflects myocardial strain secondary to the cerebrovascular event, rather than the primary cerebral event [41,48]. In terms of its temporal kinetics, it allows for the rapid identification of neurogenic cardiac injury, a phenomenon frequently accompanying severe strokes, thus offering specific value in stratifying cardioembolic etiology [25,38]. Crucially, in the context of severity and prognosis, elevated TnI levels upon hospital admission demonstrate a strong correlation with higher NIHSS scores, larger infarct volumes and higher vascular risk factors [37,49,50,51]. Moreover, TnI in the early hyperacute phase serves as a strong and standalone indicator of mortality during hospitalization, as well as of functional outcomes, in acute IS patients treated with reperfusion therapy [50,51,52,53].

#### 2.5.2. BNP and NT-proBNP

BNP and NT-proBNP serve as crucial indicators of the heart–brain axis during acute stroke, though their ability to distinguish stroke from mimics is limited in isolation due to elevations in various systemic conditions [25,41]. However, recent evidence suggests that NT-proBNP is significantly more elevated in HS than in IS, offering a potent diagnostic signal for subtype differentiation when paired with GFAP [54,55]. Crucially, NT-proBNP demonstrates superior specificity for the identification of cardioembolic etiology, with admission levels serving as a robust diagnostic, even in patients without a documented history of atrial fibrillation [37,56]. In terms of severity and prognosis, elevated baseline concentrations are strongly correlated with higher NIHSS scores and larger infarct volumes [56]. Furthermore, high admission levels of NT-proBNP act as independent predictor of 90-day mortality [56].

### 2.6. Final Considerations

Having reviewed a broad range of candidate biomarkers (Table 1), it becomes evident that no single biomarker alone currently fulfils all the requirements of the ideal acute stroke biomarker. An optimal biomarker should enable rapid stroke diagnosis, accurately differentiate IS from HS and SMs, assist in assessing the safety of reperfusion therapies, and provide reliable prognostic information. However, the available biomarkers differ considerably in their biological origin, temporal kinetics, diagnostic accuracy, and clinical applications. While some primarily reflect astroglial or neuronal injury, others provide information regarding BBB disruption, inflammation, coagulation, or systemic cardiovascular responses. Consequently, these biomarkers should be regarded as complementary rather than competitive.

This complementary nature highlights the potential of multiplexed PoC platforms capable of simultaneously measuring multiple biomarkers associated with distinct pathophysiological mechanisms. Combining biomarkers may help address diagnostic uncertainty when the information provided by an individual marker is insufficient to distinguish stroke subtypes or differentiate stroke from mimicking conditions. For example, GFAP may provide information on astroglial injury associated with haemorrhagic stroke, while complementary biomarkers may capture other aspects of the acute pathological or systemic response. The potential of a multiplexed approach is illustrated by Bustamante et al. (2017) [57] in the Stroke-Chip study, which evaluated 21 blood biomarkers in 1308 patients with suspected stroke presenting within 6 h of symptom onset. Although the biomarker panel did not improve discrimination between stroke and SMs beyond clinical variables, NT-proBNP and endostatin contributed to a model differentiating ischaemic from haemorrhagic stroke [57]. In a subsequent study involving 189 patients with stroke and an additional cohort of 62 stroke mimics, Bustamante et al. (2021) [57] further investigated a panel comprising GFAP, RBP-4, NT-proBNP, and endostatin. Combining GFAP with RBP-4 and NT-proBNP identified 51.5% of IS patients (*n* = 154) at 100% specificity. However, when SMs were included, specificity decreased to 96.8%, highlighting both the potential and the limitations of combining complementary biomarkers for early stroke differentiation [56].

By integrating biomarkers with different diagnostic and prognostic strengths, these platforms may improve stroke recognition, increase the accuracy of subtype differentiation, support patient stratification, and assist clinical decision-making during the early hyperacute phase, when rapid therapeutic intervention is critical.

However, among the biomarkers reviewed, GFAP emerges as the most promising candidate for early hyperacute stroke diagnosis. Its rapid release following intracerebral ICH, combined with its excellent ability to distinguish HS from IS and SMs, makes it uniquely suited for pre-hospital triage. Beyond its diagnostic value, GFAP also provides clinically relevant prognostic information, including the prediction of infarct volume, stroke severity, haemorrhagic transformation, and the safety of reperfusion therapies. These characteristics have established GFAP as the cornerstone biomarker for current PoC stroke diagnostics, and, therefore, it is the main focus of this review.

## 3. GFAP in Biological Fluids

Before discussing biosensing platforms for GFAP detection, it is important to consider the biological matrices in which this biomarker can be measured and their suitability for pre-hospital stroke diagnosis. Besides the analytical performance of a biosensor, the clinical applicability of GFAP detection depends on factors such as sample accessibility, collection and processing requirements, and compatibility with rapid PoC testing. Although reported GFAP concentrations can provide valuable guidance for defining the analytical sensitivity and limit of detection (LOD) required for biosensing platforms, these data are inconsistent between studies and are not equally available across all biological matrices, constraining clinical and diagnostic applicability.

Accordingly, this section discusses the main biological matrices, namely CSF, serum, plasma, whole blood, urine, saliva, and sweat, highlighting their advantages, limitations, and relevance for pre-hospital detection of GFAP for stroke diagnosis. Where appropriate, reported GFAP concentration ranges are also summarized to provide context for the analytical requirements of current and emerging biosensing technologies.

### 3.1. CSF

CSF is regarded as an attractive biological matrix for stroke biomarker research due to its proximity to the brain parenchyma, allowing it to reflect pathological processes occurring within the central nervous system [58,59,60]. Following both IS and ICH, GFAP is released into CSF as it reflects astrocytic injury, but despite these biological advantages, the clinical utility of CSF in acute stroke diagnosis remains limited [58,59,60,61]. CSF collection requires lumbar puncture, an invasive procedure that is not suitable for pre-hospital testing [58]. Nevertheless, CSF studies provide useful information regarding GFAP concentrations following cerebral ischemic injury. Brouns et al. (2010) [60] measured CSF GFAP, using an enzyme-linked immunosorbent assay (ELISA) from BioVendor, in 89 patients with IS or transient ischemic attack (TIA), with lumbar puncture performed on admission at a mean of 8.7 ± 6.2 h after symptom onset. Median GFAP concentration was 1.01 ng/mL [interquartile range (IQR): 0.76–1.66] in the stroke/TIA cohort, compared with 0.61 ng/mL [IQR: 0.45–1.06] in controls. Among patients with confirmed IS, GFAP concentrations were higher than in patients with TIA (1.10 vs. 0.83 ng/mL), and concentrations increased with both neurological severity and infarct volume, reaching median values of 1.51 ng/mL in moderate-to-severe stroke and 1.50 ng/mL in moderate-to-large infarcts. However, Brouns et al. did not investigate SMs or ICH, and GFAP concentrations did not differ according to cortical versus subcortical infarct location or TOAST-defined stroke etiology [60]. Furthermore, despite describing their cohort as hyperacute, the mean sampling time of 8.7 h does not allow these findings to be extrapolated to the first 6 h after symptom onset [58,60]. Thus, the available CSF literature provides evidence that GFAP is elevated following acute ischemic brain injury and is associated with infarct burden and stroke severity, but does not establish a GFAP concentration profile or diagnostic discriminatory performance for differentiating IS from HS or SMs during the early hyperacute phase (<6 h) [60].

### 3.2. Blood-Based Matrices

As previously discussed, astrocytic injury and BBB disruption following stroke lead to the release of GFAP into the bloodstream, making blood-based matrices the primary target for its clinical detection [37,58,61,62]. Due to their accessibility, minimal invasiveness, and compatibility with rapid diagnostic workflows, serum, plasma, and whole blood have become the most widely investigated biological matrices for GFAP quantification in acute stroke [61]. Although these matrices all originate from peripheral blood, they differ in sample preparation, biochemical composition, and analytical performance, factors that influence measured GFAP concentrations and the analytical requirements of biosensing platforms [61,63].

#### 3.2.1. Serum and Plasma

Serum and plasma represent the most extensively studied biological matrices for the detection of GFAP in the context of stroke. In serum, Jæger et al. (2023) [64] quantified GFAP using the commercial single molecule array (Simoa^®^) platform (Quanterix, Billerica, MA, USA). The study included samples collected within 4.5 h of symptom onset from 114 patients with SMs (49% females, mean age: 65 ± 17 years, stroke onset time at blood collection: 78 min [IQR: 50–140]), 131 with IS (43% females, mean age: 73 ± 13 years, stroke onset time at blood collection: 80 min [IQR: 56–126]), 21 with ICH (38% females, mean age: 70 ± 13 years, stroke onset time at blood collection: 50 min [IQR: 40–85]), and 4 with SAH (25% females, mean age: 67 ± 15 years, stroke onset time at blood collection: 141 min [IQR: 95–201]). The authors also reported the prevalence of comorbidities and previous cerebrovascular events in each patient group. These included atrial fibrillation (13–29%), diabetes (13–32%), heart disease (24–64%), hypertension (49–82%), hyperlipidemia (15–29%), smoking (8–23%), and obesity (11–30%). A history of previous acute IS, TIA, or ICH was reported in 23% of patients with IS, 33% of those with ICH, 25% of those with SAH, and 33% of those with SMs. The measuring methodology used by the authors, and available through the commercial platform, is based on sandwich immunoassays with optical detection, combining biotinylated detection antibodies with high-sensitivity horseradish peroxidase (HRP)-conjugated streptavidin and a chemiluminescent substrate, allowing for chemiluminescence intensity measurements. Median GFAP concentrations were 192 pg/mL [IQR: 109–386] in SMs, 268 pg/mL [IQR: 171–514] in IS, 1484 pg/mL [IQR: 310–5370] in ICH, and 718 pg/mL [IQR: 381–12,496] in SAH. A cutoff of 5369 pg/mL for detecting ICH yielded 25% sensitivity (95% confidence interval (CI): 11.5–43.4) and 100% specificity (95% CI: 98.6–100.0) [64].

Consistent with these findings, Luger et al. (2017) [35] assessed serum GFAP concentrations in 146 patients with IS (50% females, mean age: 74.5 ± 11.9 years, stroke onset time at blood collection: 114 ± 76 min), 45 patients with ICH (55.6% females, mean age: 68.4 ± 15.8 years, stroke onset time at blood collection: 115 ± 92 min) and 11 SM patients (45.5% females, mean age: 73.5 ± 10.0 years, stroke onset time at blood collection: 82 ± 50 min) within 6 h of symptom onset using an electrochemiluminometric immunoassay (Elecsys^®^ GFAP prototype test measured on a cobas^®^ e411 platform). The assay was modified for the study through the selection of higher-affinity monoclonal antibodies to improve analytical sensitivity. Median concentrations were 10 pg/mL [IQR: 10–10] in IS and SMs, and 160 pg/mL [IQR: 40–3270] in ICH, with a cutoff of 30 pg/mL providing an area under the curve (AUC) of 0.872 (95% CI: 0.802–0.942; *p* < 0.001) [35].

Similarly, Ren et al. (2016) [65] reported serum GFAP levels measured using a proprietary sandwich ELISA within 4.5 h of symptom onset, reporting median concentrations of 20 pg/mL [IQR: 4–80] in 79 patients with IS (37.98% females, mean age: 61.1 ± 13.33 years), 810 pg/mL [IQR: 180–3310] in 45 patients with ICH (40% females, mean age: 58.91 ± 12.18 years), 1330 pg/mL [IQR: 20–3210] in 5 patients with SAH (37.98% females, mean age: 63.08 ± 10.55 years) and 0.004 [IQR: 0.004–0.02] in 57 healthy volunteers (47.37% females, mean age: 58.93 ± 9.82 years). The GFAP assay was designed to detect both full-length GFAP and GFAP breakdown products, enabling sensitive quantification of circulating GFAP. Using a cutoff of 340 pg/mL, the AUC for differentiating ICH from SMs and IS was 0.86 (95% CI: 0.73–0.99) [65].

Comparable diagnostic performance has also been observed in plasma. Kalra et al. (2025) [66] measured plasma GFAP concentrations within 6 h of symptom onset using the commercially available i-STAT Alinity biosensing platform (with integrated electrochemical detection) in 19 patients with SMs, 258 patients with IS, and 76 patients with ICH (46.7% female, mean age: 74.6 ± 13.4 years). Median GFAP concentrations were 48 pg/mL [IQR: 29–97] in SMs, 30 pg/mL [IQR: 29–51] in IS, and 208 pg/mL [IQR: 60–5907] in ICH. A general cutoff of 55 pg/mL, as the threshold was dependent on patient age, resulted in an AUC of 0.880 (95% CI: 0.834–0.925; *p* < 0.001) for differentiating ICH from SMs and IS [66].

Han et al. (2023) [67] obtained, within 6 h of symptom onset, plasma of 51 patients with IS (60.8% females, mean age: 73 years [IQR: 59–79], stroke onset time at blood collection: 278 min [IQR: 101–633]) and 22 patients with HS (36.4% females, mean age: 55 years [IQR: 48–72], stroke onset time at blood collection: 131 min [IQR: 60–527]), measuring GFAP concentrations with the Simoa^®^ platform with chemiluminescence detection. The authors also reported patients’ medical histories, including atrial fibrillation (33.3% in IS vs. 4.5% in HS), diabetes (29.4% vs. 9.1%), dyslipidaemia (25.5% vs. 13.6%), hypertension (68.6% vs. 31.8%), and ischemic heart disease (9.8% vs. 0%). Levels were markedly higher in HS than in IS, with median concentrations of 2171.0 pg/mL [IQR: 922.8–5883] and 213.8 pg/mL [IQR: 161.8–439.4], respectively. A cutoff of 703 pg/mL provided an AUC of 0.803 (95% CI: 0.732–0.863) for distinguishing between the two conditions [67].

Finally, Katsanos et al. (2017) [36] collected plasma, within 6 h of symptom onset, of 31 patients with SMs (48.4% females, mean age: 58.1 ± 11.0 years), 121 patients with IS (48.8% females, mean age: 77.3 ± 9.1 years, stroke onset time at blood collection: 179 ± 90.8 min), 34 patients with ICH (50.0% females, mean age: 74.0 ± 9.6 years, stroke onset time at blood collection: 168 ± 74.6 min), 5 patients with SAH (20.0% females, mean age: 50.8 ± 6.2 years, stroke onset time at blood collection: 240 ± 47.4 min) and 79 controls (20.3% females, mean age: 39.5 ± 11.8 years) using a sandwich chemiluminescent immunoassay with HRP-labelled detection antibodies (Cerebral Array I Biochip on the Evidence Investigator Analyzer; Randox Laboratories Limited, Crumlin, Northern Ireland, United Kingdom). Median GFAP concentrations were 70 pg/mL [IQR: 0–240] in SMs, 110 pg/mL [IQR: 0–270] in IS, 2170 pg/mL [IQR: 550–10,120] in ICH, 0 pg/mL [IQR: 0–220] in SAH and 0 pg/mL [IQR: 0–210] in controls. A cutoff of 0.43 ng/mL yielded 91% sensitivity (95% CI: 77–98) and 97% specificity (95% CI: 93–99) for differentiating ICH from SMs, IS and SAH [36].

The variability in the results could be attributed to sampling time relative to symptom onset, analytical platforms, assay sensitivity, patient characteristics, and the specific blood matrix analyzed, and makes the direct comparison between results significantly difficult. For example, the studies included in the present comparison employed different analytical approaches, including the commercial Simoa^®^ platform, which is based on chemiluminescence, electrochemiluminometric immunoassays, chemiluminescence immunoassays, electrochemical measurements (i-STAT Alinity platform), ELISA and PoC platforms [35,36,64,65,66,67]. Consequently, absolute GFAP concentrations and diagnostic cutoffs cannot necessarily be directly compared across studies. Nevertheless, the consistent elevation of GFAP in ICH observed across both serum and plasma studies supports its use as a biological fluid for the early detection of ICH [35,36,64,65,66,67].

#### 3.2.2. Whole Blood

Despite the promising diagnostic performance demonstrated by GFAP measurements in serum and plasma, the clinical implementation of these approaches may be limited by the sample-processing requirements associated with obtaining these matrices from whole blood [61]. This consideration is particularly relevant in acute stroke, where rapid diagnosis and treatment decisions are critical and where PoC or pre-hospital testing could provide substantial clinical benefits [61].

In this context, Xue et al. (2026) [61] investigated the feasibility of measuring GFAP directly in whole blood, rather than relying on plasma, using a fully automated chemiluminescence immunoassay based on a dual-antibody sandwich principle (A6I analyser and matching reagent kits; Nanjing RealMind Biotech Co., Ltd., Nanjing, China), with the aim of simplifying sample processing and improving their accessibility for clinical applications in stroke. However, Xue et al. highlighted that whole blood is a complex matrix that introduces specific analytical challenges for immunoassays, particularly due to variability in haematocrit (HCT). HCT, defined as the proportion of blood volume occupied by red blood cells, influences the effective plasma fraction in each sample [61]. Since GFAP is in the plasma compartment, higher HCT values reduce the relative plasma volume, leading to an underestimation of the true plasma-equivalent concentration, whereas lower HCT values may result in overestimation [61]. To address this limitation, the authors implemented an HCT-based correction approach, in which individual HCT values were measured to adjust whole-blood GFAP concentrations through a calibration algorithm. GFAP concentrations were measured in matched whole-blood and plasma samples collected during the acute phase of stroke using a chemiluminescence immunoassay. The study included 52 patients with IS and 27 patients with ICH, where 66.67% were males (*n* = 52: 35 with IS, 17 with HS) and 33.33% were females (*n* = 26: 17 with IS, 10 with HS). The median age for patients with IS was 67 [IQR: 50.5–74] and those with HS was 72 [IQR: 60–78]. Median GFAP concentrations in whole blood were 78.84 pg/mL [IQR: 37.22–145.30] in IS and 549.95 pg/mL [IQR: 171.40–4239] in ICH, compared with 84.18 pg/mL [IQR: 49.64–147.25] and 582.4 pg/mL [IQR: 182.83–5219.75], respectively, in plasma. These results indicate a strong agreement between whole-blood and plasma measurements, supporting the feasibility of whole blood as an alternative matrix for GFAP detection in acute stroke [61].

#### 3.2.3. Final Considerations

Overall, the studies discussed above demonstrate the potential of GFAP as a blood-based biomarker for the early detection of ICH. Across serum, plasma, and whole-blood matrices, GFAP concentrations were consistently higher in patients with ICH than in those with IS and, where investigated, SMs, although substantial variability was observed in reported concentrations and diagnostic cutoffs. This heterogeneity reflects differences in patient populations and stroke severity, sampling time relative to symptom onset, biological matrices, analytical methods, assay characteristics, and diagnostic criteria. These factors can influence measured GFAP concentrations and the diagnostic performance of proposed cutoffs, limiting the direct comparability of results across studies. Furthermore, incomplete or inconsistently reported methodological and clinical data, including differences in patient characteristics and sampling conditions, make it difficult to determine the extent to which discrepancies reflect biological variation or methodological differences. Consequently, although the available evidence supports the diagnostic potential of GFAP in acute stroke, reported concentrations and diagnostic thresholds should be interpreted in the context of each study’s population, sampling conditions, and analytical approach rather than considered directly interchangeable.

A summary of the main studies evaluating GFAP in serum, plasma, and whole blood, including the investigated populations, sampling time, analytical methods, reported GFAP concentrations, and diagnostic performance, is presented in Table 2.

### 3.3. Exploratory Fluid Matrices

#### 3.3.1. Urine

Recent studies based on chemiluminescence detection have investigated urine as a potential biological matrix for the detection of GFAP in stroke, offering a completely non-invasive alternative for biomarker assessment [62,68,69]. Using the commercial ultrasensitive Simoa^®^ assay, Kohlhase et al. (2023) [69] detected urinary GFAP in patients sampled within 96 h of stroke onset, distinguishing stroke (184.7 ± 362.4 pg/mL) from SMs (27.3 ± 24.1 pg/mL) [69]. Using the same analytical platform and time constraint, a study by Lieschke et al. (2025) [68] reported that urinary GFAP was the strongest predictor of in-hospital functional outcome among the biomarkers evaluated, suggesting potential prognostic rather than diagnostic utility. This prognostic value was evidenced by significant concentration differences between patients with favourable outcomes and those who died (Δ = 13.45 pg/mL at discharge, *p* = 0.02), alongside an optimal urinary cutoff of 26.6 pg/mL for predicting 3-month mortality (69.2% sensitivity, 72.5% specificity) [68]. However, urinary GFAP concentrations were unable to differentiate IS from ICH, limiting their diagnostic value for acute stroke classification [62,68].

Despite these encouraging findings, urinary GFAP has not yet been evaluated during the early hyperacute phase of stroke, when rapid diagnosis is essential for pre-hospital decision-making [62,68]. Consequently, the current evidence is insufficient to support urine as a suitable biological matrix for PoC stroke diagnosis in pre-hospital settings, and further studies are required to establish its clinical applicability.

#### 3.3.2. Saliva

Saliva has attracted interest as a completely non-invasive biological matrix for stroke biomarker detection due to its ease of collection and potential compatibility with PoC testing [62]. However, to date, no clinical studies have demonstrated the diagnostic utility of salivary GFAP in patients with acute stroke [62]. Consequently, despite its attractive characteristics for pre-hospital testing, saliva remains an experimental matrix, and further studies are required to determine whether GFAP can be reliably detected at clinically relevant concentrations and within the early hyperacute time window necessary for stroke diagnosis [62].

#### 3.3.3. Sweat

Sweat has been explored as a non-invasive biological fluid for PoC applications due to its compatibility with wearable biosensing technologies [70,71]. However, similar to saliva, no studies have investigated sweat as a biological matrix for the detection of GFAP in patients with acute stroke. Consequently, there is currently no evidence supporting the use of sweat for GFAP-based stroke diagnosis, and its applicability in pre-hospital PoC settings remains to be demonstrated.

### 3.4. Final Considerations

Overall, the suitability of biological matrices for GFAP detection in pre-hospital stroke diagnosis depends not only on the biomarker’s detectability and diagnostic performance, but also on sample accessibility, processing requirements, and compatibility with rapid PoC testing. Among the matrices reviewed, serum and plasma currently have the strongest clinical evidence supporting the use of GFAP for the early identification of ICH, as multiple studies have reported higher concentrations in patients with ICH than in those with IS and SMs. However, differences in reported GFAP concentrations and diagnostic cutoffs across studies highlight the influence of sampling time, patient characteristics, biological matrix composition, and analytical methodology, emphasizing the need for standardized measurement procedures and further clinical validation.

Whole blood also represents a promising alternative for pre-hospital applications, as it may reduce sample-processing requirements and facilitate rapid PoC testing. In contrast, although CSF provides direct insight into central nervous system injury, its invasive collection makes it unsuitable for routine pre-hospital testing. Similarly, urine, saliva, and sweat offer potential advantages in terms of non-invasive collection, but the available evidence remains insufficient to establish their diagnostic utility during the early hyperacute phase of stroke. In particular, the absence of clinical studies evaluating GFAP in saliva and sweat, together with the limited diagnostic discrimination demonstrated by urinary GFAP, currently restricts their applicability in this context.

## 4. Biosensors’ Review

**Figure 1:**
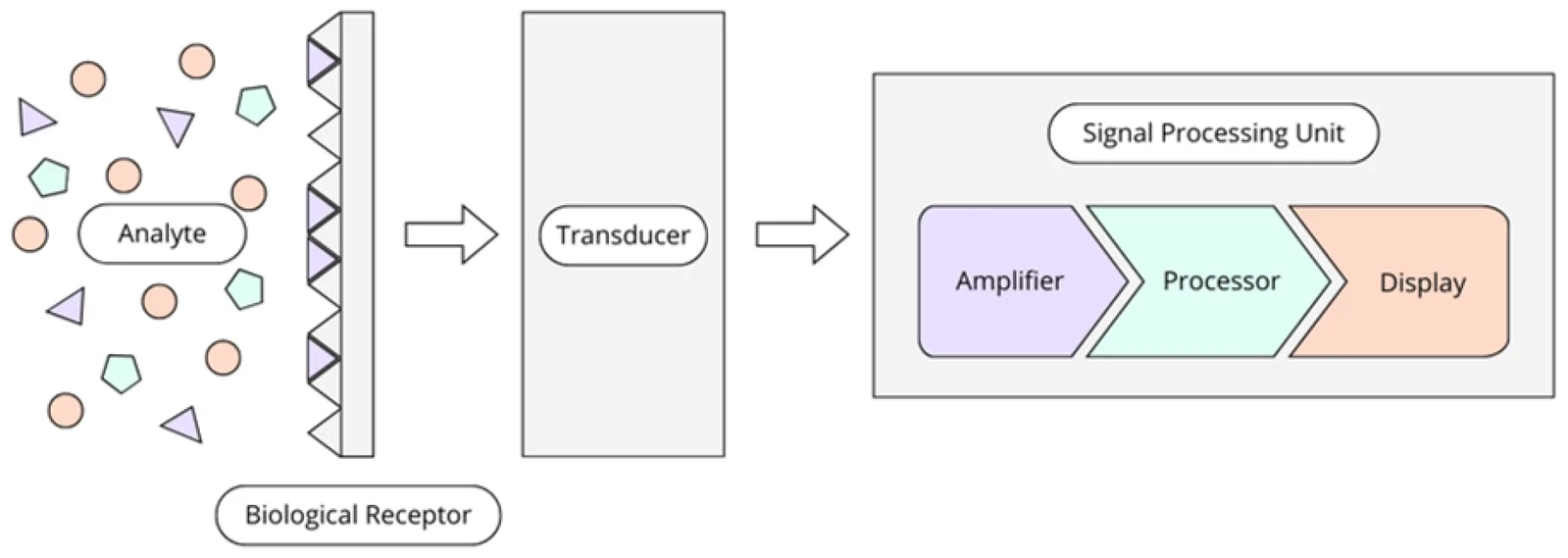
The basic components of a biosensor, including the bioreceptor, transducer, signal processor, and output signal. Redrawn and adapted from Tetyana et al. (2021) [74] under the Creative Commons Attribution 3.0 International (CC BY 3.0) licence.

Biosensors are analytical systems in which a biological recognition component is coupled to a physical transducer to produce a quantifiable signal [72,73]. These are recognized for having great selectivity and sensitivity, with quick response, and being user friendly and portable [72,73]. As illustrated in Figure 1, which shows a simplified view of a biosensor architecture, a biosensor is composed of three principal elements: a biological receptor, responsible for selectively recognizing and binding the target analyte; a transducer, which converts the biochemical event resulting from analyte recognition into a quantifiable electrical signal; and a signal processing unit, which amplifies, processes, and decodes the signal to produce meaningful analytical information [72,73].

Biosensors can be classified by their transduction mode into electrochemical, mechanical and optical biosensors [72,75]. Electrochemical biosensors convert biochemical interactions into electrical signals, for example changes in current (amperometric), potential (potentiometric) or impedance (impedimetric) that arise from enzymatic reactions, affinity binding or redox events [76,77]. Mechanical biosensors detect changes in mass, surface stress or resonant frequency induced by target binding at a functionalized surface [78,79]. Optical biosensors detect the interaction between the biorecognition element and optical field by monitoring changes in light, such as intensity (amplitude), phase, wavelength (spectral shift) or angle of incidence/reflection [75,80].

This chapter reviews different biosensors developed for the detection of GFAP, focusing on their intended applications and assessing, where appropriate, their potential applicability to stroke diagnosis, even when originally designed for other clinical purposes.

### 4.1. Biosensors for GFAP Quantification

Significant research efforts have been directed towards the development of selective, sensitive, and clinically viable biosensors for GFAP detection. Different transduction mechanisms have been explored to convert the specific recognition of GFAP into measurable signals, including electrochemical, mechanical, optical, and other emerging approaches [81,82,83,84,85,86,87,88,89,90,91,92,93,94,95,96,97,98,99]. These platforms differ considerably in terms of sensing principles, analytical performance, biological matrices, assay duration, and degree of integration, reflecting the different strategies investigated to achieve rapid and reliable GFAP detection [81,82,83,84,85,86,87,88,89,90,91,92,93,94,95,96,97,98,99]. The following sections summarize the main biosensor approaches reported for GFAP detection, with particular emphasis on their sensing mechanisms and their potential applicability to PoC and early hyperacute stroke diagnosis.

#### 4.1.1. Literature Search Methodology

A literature search was conducted to identify recent studies on GFAP biosensors and their potential applications in rapid and point-of-care detection. Searches were performed using PubMed and Scopus, with combinations of the terms “GFAP”, “glial fibrillary acidic protein”, “biosensor”, “biosensing”, and “point-of-care”. The search aimed to identify, as comprehensively as possible, the different sensing and detection strategies reported for GFAP during the last five years (publications before 2020 were excluded). Although the review focuses on stroke, the search was not restricted to stroke-related studies, allowing the inclusion of GFAP biosensors developed for other neurological conditions. Studies were selected based on their relevance to GFAP detection, diversity of sensing strategy, analytical performance, and potential clinical or point-of-care applicability. When multiple studies employed similar detection strategies, preference was given to studies providing distinctive approaches, representative technological advances, or greater relevance to the scope of the review, to avoid unnecessary repetition and provide a broader overview of the field. Studies with limited relevance to GFAP biosensing or the objectives of the review were excluded. Relevant studies identified through supplementary literature searches, related publications, and reference lists were also considered. The search and subsequent selection process resulted in 19 studies being included in this section.

#### 4.1.2. Electrochemical Biosensors

Electrochemical biosensors have been extensively investigated for GFAP detection because they can convert biomolecular recognition events into measurable electrical signals through changes in current, potential, or impedance [81,82,83,84,85,86,87]. Their compatibility with miniaturized electrodes, relatively simple instrumentation, and potential for integration with microfluidic and portable systems makes them particularly attractive for PoC applications. The studies described below explore different electrochemical transduction mechanisms, electrode modifications, recognition strategies, and biological matrices for GFAP detection.

**Figure 2:**
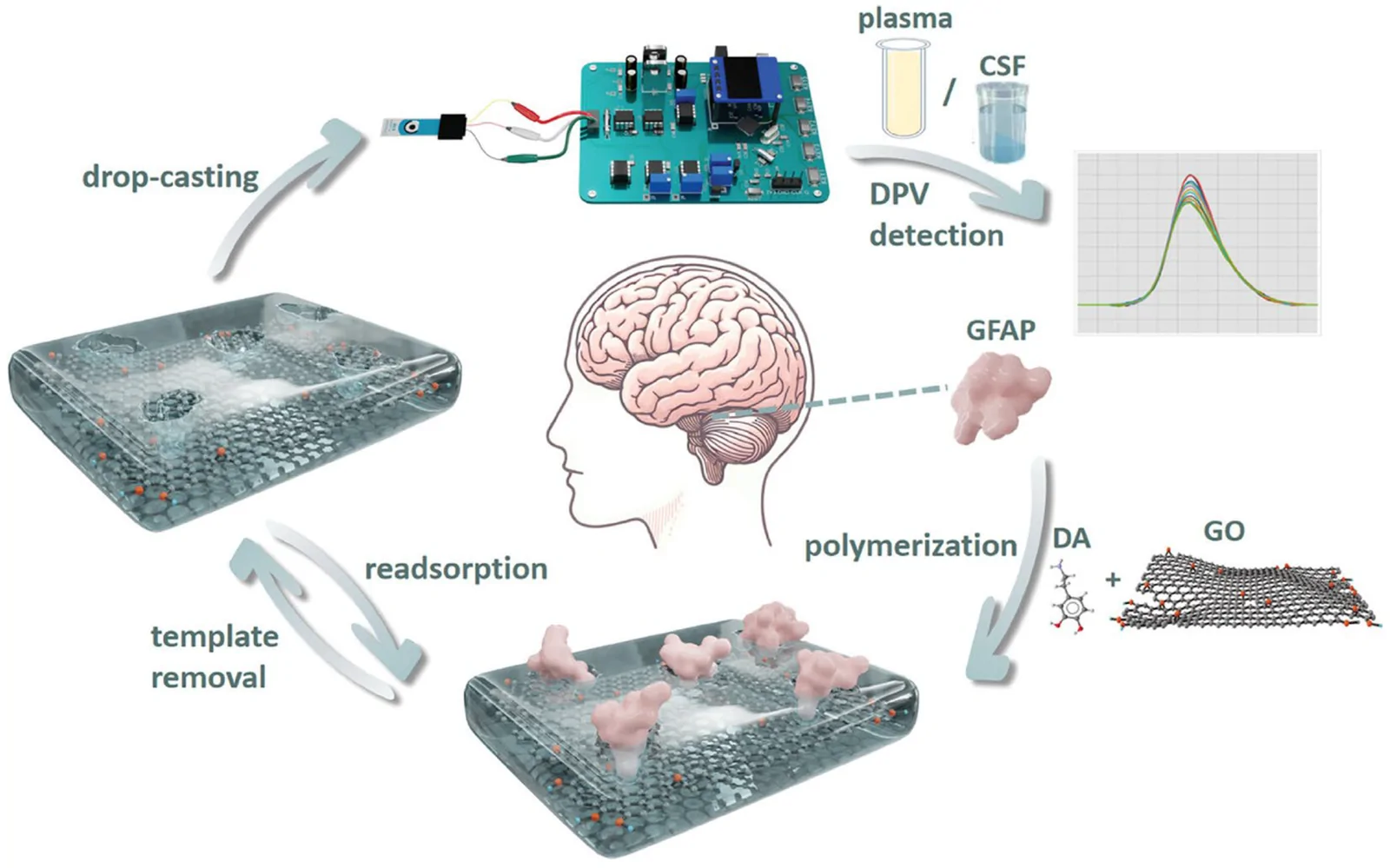
MIP-based PoC sensing platform for GFAP detection. Illustration of the synthesis of the rGO/PDA-MIP composite and its application for GFAP detection using a miniaturized PoC electrochemical readout platform. Reproduced from Li et al. [81] *Advanced Healthcare Materials* (2024), under the applicable Creative Commons licence.

Li et al. (2024) [81] developed an ultrasensitive electrochemical biosensor based on a reduced graphene oxide (rGO)/polydopamine (PDA) molecularly imprinted polymer (MIP) (rGO/PDA-MIP) for the quantification of GFAP in plasma or CSF, with potential applications in the diagnosis and monitoring of neurological diseases (Figure 2). In this approach, GFAP was initially used as a template molecule during formation of the MIP, generating specific cavities capable of selectively recognizing GFAP. The sensing layer was coupled to a miniaturized PoC readout based on differential pulse voltammetry (DPV), allowing the interaction between GFAP and the imprinted polymer to be converted into an electrochemical signal. The platform achieved an ultra-low LOD of 754.5 ag/mL and a linear detection range (LDR) from 1 fg/mL to 1 ng/mL using GFAP diluted in phosphate-buffered saline (PBS). Importantly, the sensor was also evaluated using three clinical plasma samples (37 to 56,424 pg/mL), showing good agreement with Simoa^®^ measurements, and a sample-to-result time of approximately 10 min was reported [81].

Ozcelikay et al. (2022) [82] developed a label-free impedimetric immunosensor using a screen-printed carbon electrode modified with L-cysteine and gold nanoparticles for the ultrasensitive detection of GFAP, in serum, aiming for the monitoring of central nervous system injuries. Anti-GFAP antibodies were covalently immobilized on the electrode surface, and GFAP binding to the capture antibodies altered the electron-transfer resistance of the electrode. This change was measured by electrochemical impedance spectroscopy using a redox probe, allowing GFAP concentration to be determined without the need for an additional detection label. The sensor achieved an LOD of 51.0 fg/mL and an LDR of 1–1000 pg/mL, under optimum conditions using a standard solution, and an analytical sensitivity of 0.308 Ω/(pg/mL) in synthetic serum, together with good selectivity, repeatability, and stability for more than 15 days and an assay time of 1 h [82].

**Figure 3:**
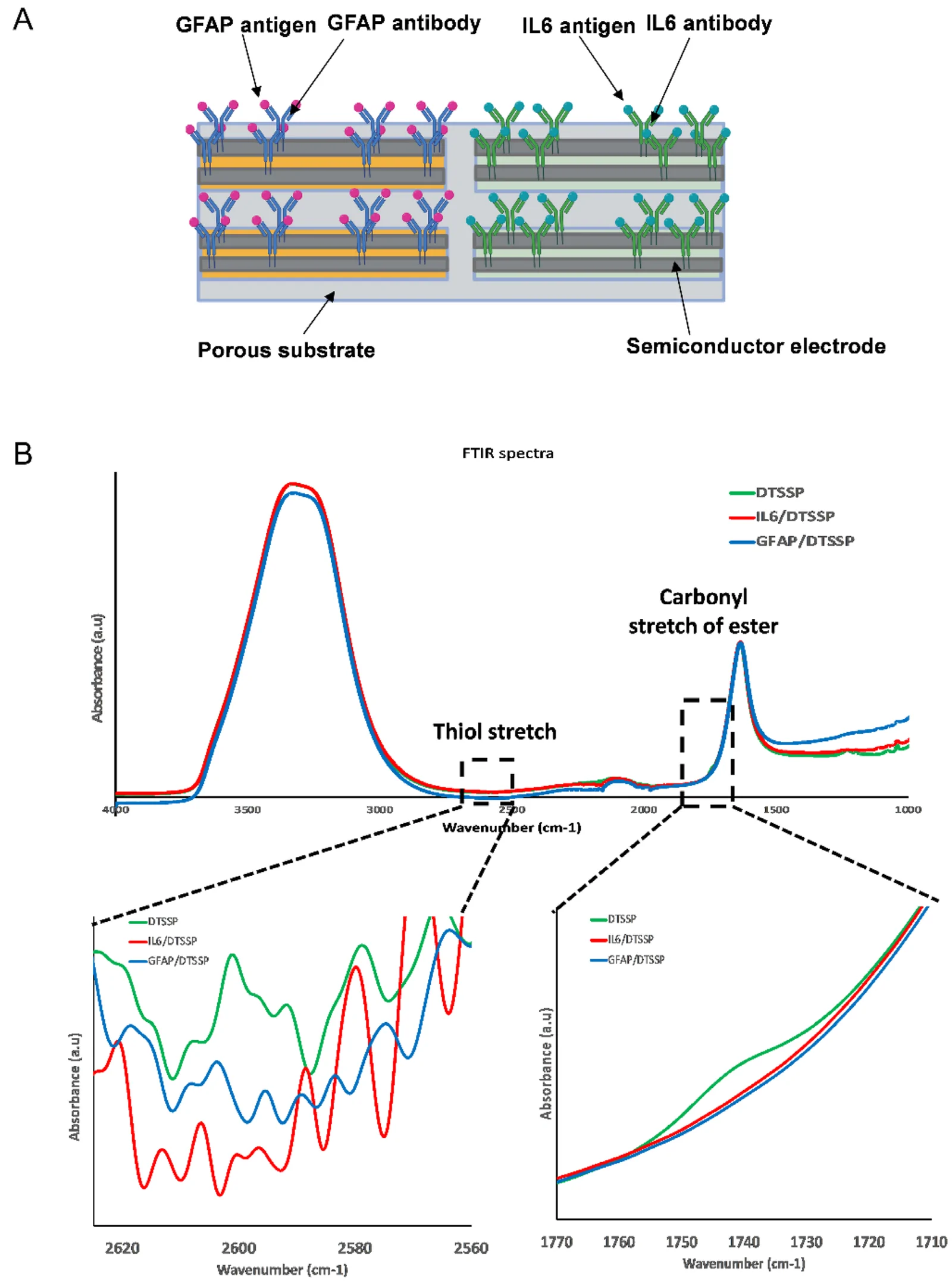
Schematic representation of the ZnO-based immunosensor and its surface functionalization: (**A**) sensor design and surface chemistry; (**B**) FTIR characterization of DTSSP, GFAP antibody, and IL-6 antibody immobilized on the ZnO surface. Reproduced from Shahub et al. (2022) [83] under the Creative Commons Attribution (CC BY) licence.

A different approach was proposed by Shahub et al. (2022) [83], who developed a portable and automated multiplexed electrochemical impedance immunosensor for the simultaneous detection of GFAP and IL-6 in patients with traumatic brain injury (TBI) using sweat, as it can be observed in Figure 3. The platform consists of a wearable strip containing multiple pairs of functionalized electrodes, with each electrode pair containing capture antibodies specific to the target biomarker. The binding of GFAP to the corresponding antibodies produces changes in impedance, which are converted into biomarker concentrations by the integrated sensing system. A portable potentiostat and Bluetooth-enabled wireless communication were incorporated into the platform, enabling continuous monitoring. For GFAP, the sensor demonstrated an LOD of 14 pg/mL over a concentration range of 7.81–2664 pg/mL with a sweat analogue spiked with GFAP [83].

Salahandish et al. (2022) [84] developed a portable electrochemical impedance immunosensor for the detection of TBI, mild TBI, spinal cord injury, and stroke using plasma as the biological matrix. The platform uses a self-powered microfluidic device to automate sample handling, reagent delivery, and electrochemical detection. Anti-GFAP antibodies were covalently immobilized on the working electrode, and direct binding of GFAP was detected through changes in impedance. The system achieved an LOD of 3 pg/mL and an LDR of 10–1000 pg/mL, with a sample-to-result time of less than 30 min. Furthermore, a plasma sample, from a healthy individual, was spiked with three different concentrations of GFAP (50, 100, and 500 pg/mL), which the biosensor was able to distinguish. The integration of microfluidic sample handling with the electrochemical sensor represents an important step towards reducing manual operations and developing compact PoC systems [84].

García-Rodrigo et al. (2024) [85] developed an amperometric immunosensor for GFAP detection in CSF using an rGO/molybdenum disulfide-silver nanoparticle (rGO/MoS_2_-AgNP) nanocomposite-modified screen-printed carbon electrode. Anti-GFAP antibodies were covalently immobilized on the electrode, and GFAP binding was monitored through changes in the electrocatalytic reduction of hydrogen peroxide, enabling label-free amperometric detection. The nanocomposite electrode provided a surface suitable for biomolecular recognition and electrochemical signal generation, reaching an LOD of 160 pg/mL and an LDR of 600 pg/mL to 100 ng/mL, using GFAP standard solutions diluted in PBS. The sensor demonstrated good sensitivity, specificity, selectivity, reproducibility, and repeatability, with stability for up to 23 days. Furthermore, the sensor showed good response to three samples of CSF from encephalitis patients (6 × 10^3^, 27 × 10^3^ and 38 × 10^3^ pg/mL); however, the assay time was 2 h 30 min long [85].

**Figure 4:**
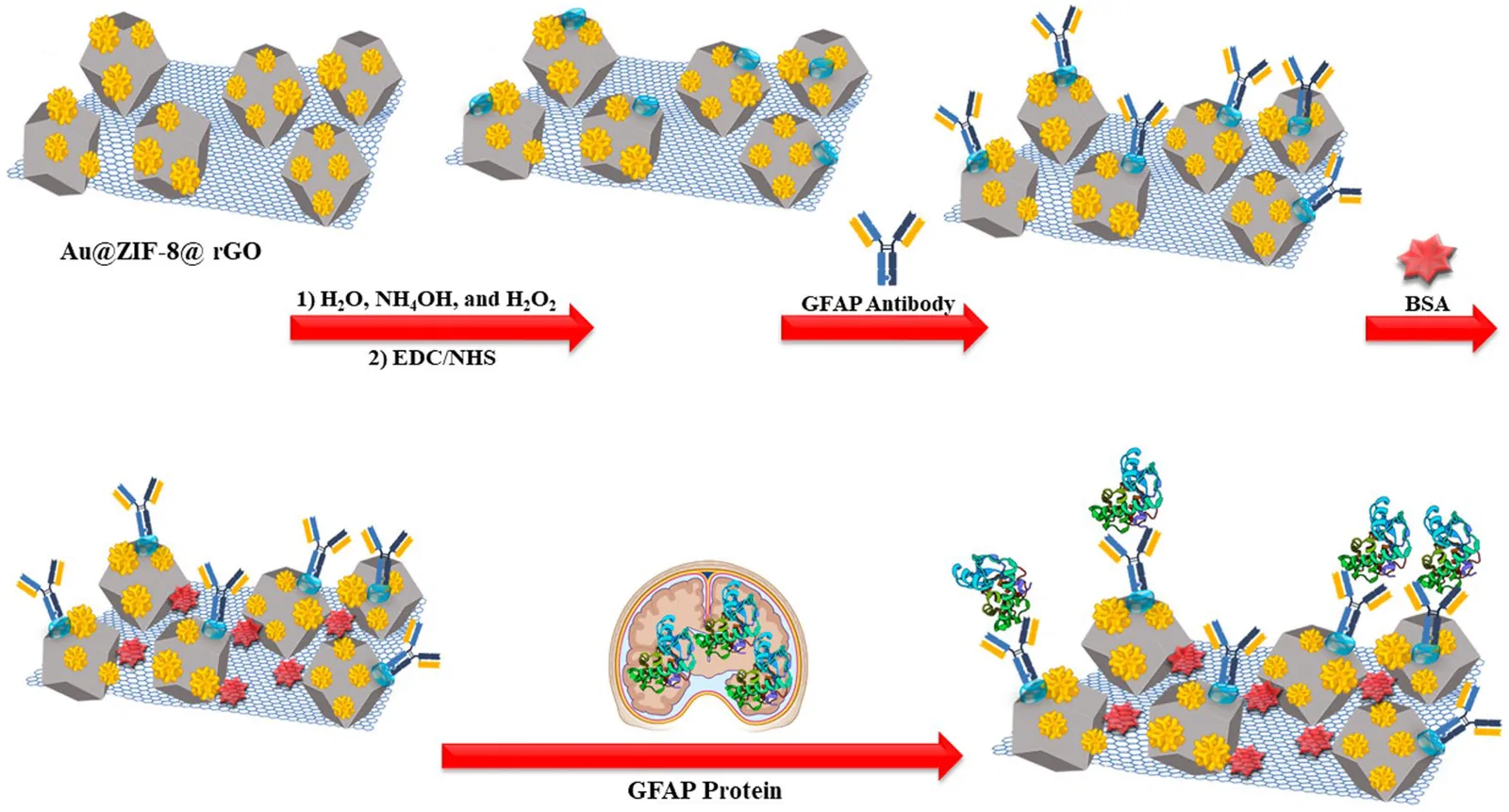
Schematic illustration of the preparation of the GFAP-bovine serum albumin (BSA)-anti-GFAP-Au@ZIF-8@rGO/SPE electrochemical immunosensor for GFAP detection. Reproduced from Mehmandoust et al. (2022) [86] under the Creative Commons Attribution (CC BY 4.0) licence.

Mehmandoust et al. (2022) [86] extended electrochemical GFAP detection to urine by developing a label-free impedimetric immunosensor based on a metal–organic framework composite for TBI diagnosis. The sensing platform consisted of Au@ZIF-8@rGO, onto which anti-GFAP antibodies were covalently immobilized (Figure 4). When GFAP binds to the capture antibodies, the electrode surface becomes increasingly blocked, producing an increase in charge-transfer resistance that can be measured by electrochemical impedance spectroscopy. The sensor achieved an LOD of 50 fg/mL, using GFAP standard solutions diluted in PBS, and showed good specificity, selectivity, reproducibility, and repeatability, while also presenting potential advantages in terms of portability, cost, and user-friendliness. Additionally, the sensor was tested using three diluted urine samples in PBS with added GFAP (0.1, 0.2, 3.5 pg/mL), presenting good results; conversely, the assay time was longer than 45 min [86].

**Figure 5:**
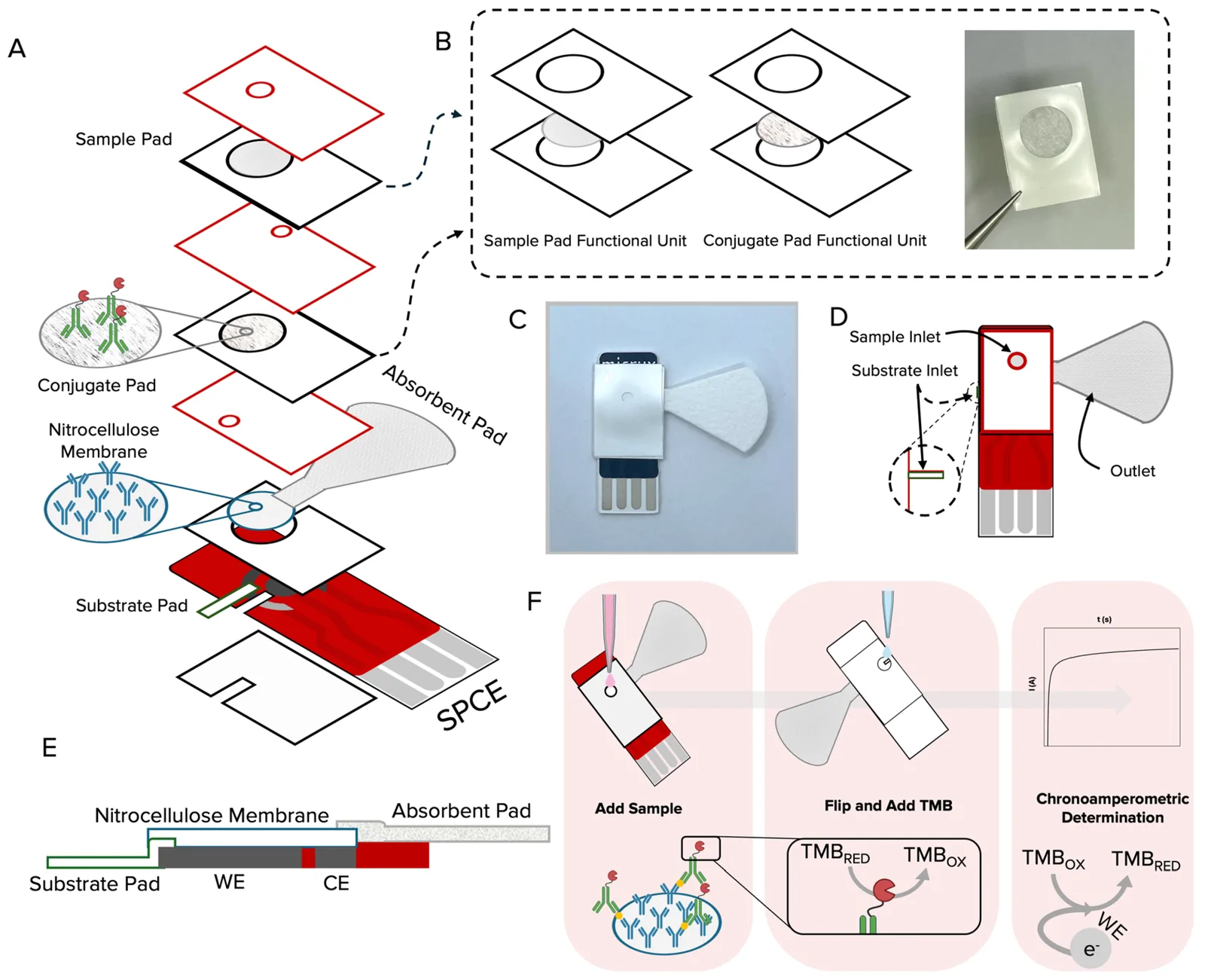
Design and operation of an eVFIA device for GFAP detection: (**A**) multilayer architecture of the device; (**B**) sample and conjugate structural units; (**C**) fabricated device; (**D**) main and auxiliary inlets and outlet; (**E**) cross-sectional arrangement of the substrate pad, nitrocellulose membrane, screen-printed carbon electrode (SPCE), and absorbent pad; (**F**) workflow of the eVFIA assay and electrochemical detection of GFAP. Reproduced from Rioboó-Legaspi et al. (2026) [87] under the Creative Commons Attribution 4.0 International (CC BY 4.0) licence.

More recently, Rioboó-Legaspi et al. (2026) [87] developed an electrochemical vertical-flow immunoassay (eVFIA) specifically aimed at rapid and decentralized GFAP detection for stroke assessment. The platform integrates a multilayer paper-based microfluidic device with a screen-printed carbon electrode (Figure 5A). GFAP is captured using immobilized antibodies and subsequently recognized by HRP-labelled detection antibodies, forming a sandwich immunoassay (Figure 5A,B). The HRP-mediated enzymatic reduction of 3,3′,5,5′-tetramethylbenzidine generates an electrochemical signal that is quantified by chronoamperometry, as presented in Figure 5F. The platform achieved an LOD of 85 pg/mL and an LDR of 0.1–10 ng/mL using a blocking solution (0.5% Tween 20 with 1% BSA in 10 mM PBS, pH 7.4) and demonstrated good selectivity against MMP-9 and S100B. Importantly, four diluted (1:10 in 10 mM PBS 7.4) clinical serum samples (280, 1.6 × 10^3^, 4.74 × 10^3^ and 12.2 × 10^3^ pg/mL) from stroke patients were successfully analyzed, with results showing statistical concordance with ELISA. The platform required approximately 10 min from serum addition to readout and incorporated a 3D-printed casing and USB-C portable potentiostat, highlighting its potential for decentralized testing [87].

Overall, these studies demonstrate the versatility of electrochemical platforms for GFAP detection. Different transduction mechanisms, including impedance, amperometry, and DPV, have been successfully combined with antibody-based recognition or MIPs. The use of screen-printed electrodes, nanostructured electrode surfaces, microfluidic systems, and portable readout devices further supports the potential of electrochemical biosensors for miniaturized and PoC applications. The sensors also showed great sensitivity overall and some were able to reach the fg/mL or even ag/mL range.

#### 4.1.3. Mechanical Biosensors

Mechanical biosensors detect biomolecular interactions by converting changes in mechanical properties, such as mass or resonance frequency, into a measurable signal [88,89]. For GFAP detection, surface acoustic wave (SAW) and quartz tuning fork (QTF)-based platforms have been investigated as label-free approaches, in which the binding of GFAP to surface-immobilized antibodies produces changes in the mechanical response of the transducer [88,89].

Agostini et al. (2021) [88] developed a SAW-based mechanical immunosensor for the detection of GFAP in serum to detect multiple brain pathologies. The authors investigated three antibody immobilization strategies based on antifouling properties, antibody orientation, and surface coverage, with the antifouling strategy providing the best performance. This strategy was subsequently implemented on an ultrahigh-frequency SAW lab-on-chip for direct and label-free GFAP detection. The platform consisted of an ultrahigh-frequency SAW device mounted on a printed circuit board and integrated with a polydimethylsiloxane microfluidic channel for sample handling. Binding of GFAP at the sensor surface produces changes in the acoustic response of the SAW device, enabling its detection without the use of additional labels. The resulting platform demonstrated high sensitivity, with an LOD of 35 pM in a sample of diluted GFAP in PBS and in serum-matrix (BSA and fetal bovine serum, both diluted in PBS), as well as good specificity, selectivity, and repeatability. The integration of the SAW device with microfluidics also demonstrated potential for portability and automation [88].

**Figure 6:**
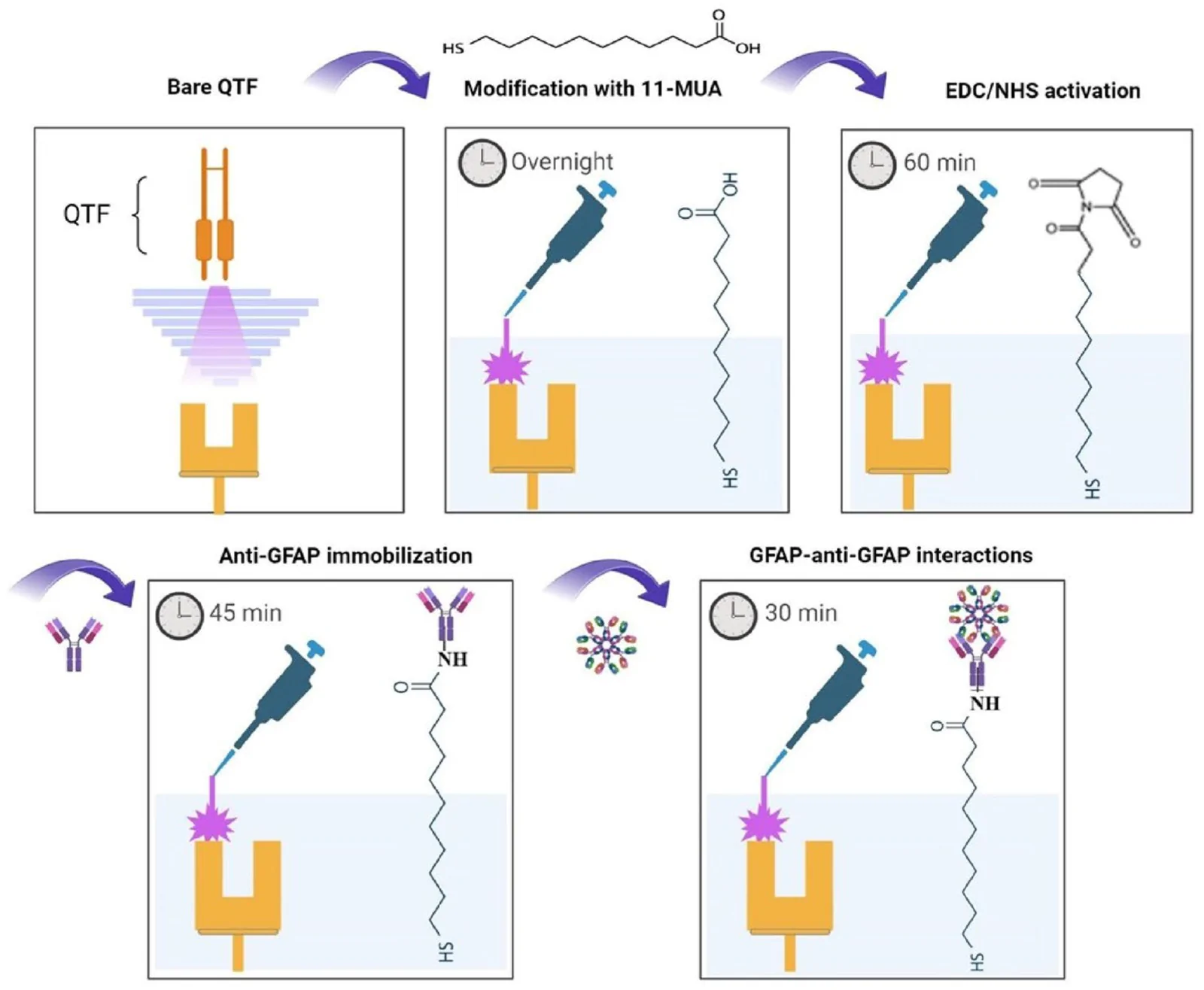
Schematic representation of the immobilization strategy of the 11-mercaptoundecanoic acid (11-MUA)-functionalized QTF biosensor for GFAP detection. Reproduced from Özcan et al. (2025) [89] under the applicable Creative Commons licence.

More recently, Özcan et al. (2025) [89] developed a mass-sensitive QTF-based immunosensor for the diagnosis of TBI in serum (Figure 6). The gold-coated QTF surface was functionalized with 11-mercaptoundecanoic acid (11-MUA), which enabled the covalent immobilization of anti-GFAP antibodies. In this case, GFAP binding to the antibody-functionalized surface increases the effective mass of the QTF and consequently produces a shift in its resonance frequency. This frequency shift was measured using an Owon Smart DS5032E oscillator and correlated with the GFAP concentration, enabling direct and label-free detection. The biosensor demonstrated a sensitivity of 0.2729 Hz/(fg/mL), an LOD of ≤0.05 fg/mL and an LDR of 0.05–25 fg/mL (using phosphate buffer (PB)), together with good specificity, selectivity, reproducibility, and repeatability. The sensor was tested with commercial serum samples diluted in PB (*n* = 3), with each one spiked with two different concentrations of GFAP (1 × 10^−3^ and 10 × 10^−3^ pg/mL), showing good results. The use of QTF also provides potential for developing a low-cost and portable GFAP detection platform; however, the sample-to-result time was longer than 30 min [89].

Overall, these studies demonstrate that mechanical transducers can provide highly sensitive, label-free GFAP detection while offering potential advantages in terms of miniaturization and portability. However, current evidence remains limited to a small number of studies, and important parameters such as long-term stability, complete analytical ranges, and assay duration are not consistently reported.

#### 4.1.4. Optical Biosensors

Optical biosensors detect biomolecular interactions by monitoring changes in an optical signal, such as fluorescence, photoluminescence, refractive index, absorbance or other light-dependent properties [75,80]. Different optical strategies have been investigated, including upconversion nanoparticles (UCNPs), optical interferometry, electrochemiluminescence (ECL), plasmonic fluorescence, and fluorescence-based amplification approaches.

Kakkar et al. (2025) [90] developed an optical UCNP-based immunosensor for GFAP detection in serum, with the aim of differentiating stroke from SMs. The sensing platform employed NaYF_4_ nanoparticles doped with Yb^3+^ and Er^3+^, which convert near-infrared (NIR) excitation into visible photoluminescence. The UCNPs were functionalized with anti-GFAP antibodies, and direct binding of GFAP to the antibody-functionalized nanoparticles resulted in quenching of the UCNP photoluminescence. The decrease in the optical signal was correlated with the GFAP concentration, enabling label-free detection. The sensor achieved an LOD of 0.082 pg/mL and an LDR of 10–5000 pg/mL in 20% human serum (*n* = 8: 5–5 × 10^3^ pg/mL) in the absence of other biomarkers, together with high sensitivity, specificity, and selectivity. However, the platform currently relies on laboratory optical equipment, including an NIR laser and spectrometer, is neither portable nor automated, and has a long assay time of more than 2 h [90].

Xiao et al. (2025) [91] developed an optical microfiber interferometric immunosensor for GFAP detection in serum and CSF to diagnose TBI. In this platform, anti-GFAP antibodies were covalently immobilized into a gold nanostar-coated optical microfiber. The binding of GFAP changes the local refractive index at the microfiber surface, which is detected through the strong evanescent optical field surrounding the microfiber. This interaction produces a shift in the optical spectrum, with a single GFAP molecule producing a reported shift of 0.06 nm. The sensor achieved ultra-low LODs of 0.21 aM in 10% fetal bovine serum, 0.10 aM in 10% artificial CSF, and 0.09 aM in PBS, while an LDR from 1 aM to 0.1 nM was obtained in PBS. These results demonstrate extremely high sensitivity, 0.324 nm/log(M) in 10% artificial CSF and 0.305 nm/log(M) in 10% foetal bovine serum, achievable using optical interferometric detection, although the assay time has not yet been established and further development is required for automation and practical implementation [91].

Han et al. (2025) [92] developed an ECL immunosensor for the diagnosis of TBI using serum. The platform uses a RuMoNi-based ECL emitter in combination with a sandwich immunoassay. GFAP is first captured by primary antibodies and subsequently recognized by secondary antibodies conjugated to a PtCuCo nanomaterial. The binding of GFAP brings the PtCuCo nanomaterial into proximity with the ECL emitter, resulting in quenching of the emitted light. The decrease in ECL intensity is therefore proportional to the GFAP concentration. The sensor achieved an LOD of 1.66 pg/mL and a dynamic range of 0.005–100 ng/mL, together with high sensitivity (812.59 a.u./log(ng/mL)) using GFAP standard solutions diluted in PBS, specificity, selectivity, reproducibility, and repeatability. Furthermore, the sensor was tested using a sample of serum spiked with different concentrations of GFAP (*n* = 4: 510, 890, 1.37 × 10^3^ and 1.86 × 10^3^ pg/mL), showing good promise for clinical utility (relative standard deviation (RSD) < 5%). Despite this analytical performance, the platform is not currently portable or automated and requires more than 2 h for the assay [92].

Another optical approach was reported by Zheng et al. (2022) [93], who developed a plasmonic NIR fluorescent immunosensor for GFAP detection in plasma to diagnose TBI. The platform uses a gold nanopyramid array functionalized with capture antibodies together with a DyLight 755-labelled anti-GFAP detection antibody. GFAP binding therefore produces a sandwich complex at the gold nanopyramid surface, enhancing the NIR fluorescence signal. The resulting fluorescence is measured using a portable fluorescence reader, providing a more compact optical detection configuration. The sensor demonstrated a sensitivity of 127 a.u./log(pg/mL), an LOD of 0.6 pg/mL, and an LDR of 1–500 pg/mL in commercial plasma spiked with GFAP (*n* = 13: 1.0 to 100 × 10^3^ pg/mL), in addition to good specificity and selectivity. However, the assay requires more than 1 h and additional plasma preparation, while the reported detection range may not fully cover the GFAP concentrations expected in stroke [93].

Indongo et al. (2025) [94] developed a fluorescence-based GFAP biosensor using copper nanoclusters (CuNCs) and a graphene oxide (GO) biosensor for the diagnosis of glioblastoma using plasma, urine, or saliva. Anti-GFAP antibodies were conjugated to CuNCs, while GO acted as a fluorescence quencher through Förster resonance energy transfer (FRET). In the absence of GFAP, the close interaction between CuNCs and GO results in fluorescence quenching. Upon GFAP binding, this interaction is disrupted, reducing FRET and restoring the fluorescence signal, thereby producing a “turn-on” response proportional to GFAP concentration. The sensor achieved an LOD of 32 pg/mL and an LDR of 0–46 ng/mL, together with high specificity and selectivity, by adding incremental volumes of purified GFAP antigen to the probe solution. Importantly, the authors also incorporated the sensing system into a paper-strip format, demonstrating qualitative detection of GFAP in biological samples spiked with GFAP under UV illumination. This format highlights the potential for simple and low-cost optical testing, although the strips currently provide only qualitative detection and further optimization is required [94].

A multiplexed optical biosensor was developed by Wang et al. (2025) [95], who reported an ultrasensitive surface-enhanced Raman scattering (SERS) immunosensor for the simultaneous detection of GFAP and retinol-binding protein 4 (RBP4) in serum for stroke subtype discrimination. The sensing platform is based on GO-supported Au-Ag roughened nanoboxes (GO@AuAgRNB), which provide abundant high-density SERS hotspots through electromagnetic and chemical enhancement. The nanocomposite was combined with magnetic beads to form a sandwich immunoassay, in which GFAP- and RBP4-specific antibodies were conjugated to the SERS probes, while capture antibodies were immobilized on the magnetic beads. Different Raman reporters were used for GFAP and RBP4, enabling their simultaneous and independent quantification through their characteristic Raman peaks. The platform achieved an LDR of 0.1 pg/mL–0.1 μg/mL and an LOD of 0.16 pg/mL for GFAP. The sensor demonstrated good specificity and selectivity in spiked serum samples. Furthermore, the SERS substrate showed good stability for more than 30 days, while the complete assay required approximately 30 min and successfully analyzed fifty clinical serum samples diluted 1000-fold, comprising twenty-five IS (mean concentration: 0.26 ng/mL) and twenty-five HS patients (mean concentration: 12.41 ng/mL). The platform enabled discrimination between stroke subtypes with a cutoff of 2.07 ng/mL, which yielded an AUC of 0.998. However, the platform is not yet automated or portable, and its reproducibility and repeatability need to be established [95].

Finally, Cao et al. (2026) [96] developed a proof-of-concept fluorescent biosensor based on isothermal nucleic-acid amplification for the early detection of Alzheimer’s disease using serum. Unlike most of the antibody-based platforms described above, the sensor uses GFAP-specific deoxyribonucleic acid (DNA) aptamers immobilized on streptavidin-functionalized magnetic nanoparticles. GFAP binding releases complementary DNA strands, which initiate an exponential amplification reaction followed by a hybridization chain reaction. This amplification separates a fluorescent reporter from its quencher, generating a fluorescence signal proportional to the GFAP concentration. The platform achieved an LOD of 0.24 pg/mL and an LDR of 10 pg/mL–10 µg/mL using GFAP-spiked human serum, together with good specificity, selectivity, reproducibility, and repeatability. To further assess its clinical utility, the sensor was evaluated using 12 clinical serum samples, comprising six samples from patients with Alzheimer’s disease and six from cognitively normal individuals. GFAP concentrations measured with the proposed sensor ranged from 88 to 127 pg/mL in the cognitively normal samples and from 235 to 358 pg/mL in the Alzheimer’s disease samples, with significantly higher concentrations observed in the Alzheimer’s disease group. These results provided preliminary clinical evidence supporting the ability of the sensor to discriminate between AD and cognitively normal individuals, although the small clinical cohort limits the strength of this evidence and warrants further validation in larger patient populations [96].

Overall, optical biosensors demonstrate considerable versatility for GFAP detection, ranging from nanoparticle-based photoluminescence and fluorescence to interferometric and electrochemiluminescent detection. Several platforms achieve very low detection limits, while approaches such as plasmonic fluorescence, paper-strip detection, and optical microfibers demonstrate potential for miniaturization and simplified detection. Nevertheless, many of the reported systems still rely on laboratory-based optical instrumentation, relatively long assay times, or additional sample-processing steps. Moreover, several platforms have been evaluated primarily using serum, plasma, CSF, or spiked samples, meaning that further clinical validation is required to establish their suitability for rapid GFAP detection in acute stroke.

#### 4.1.5. Emerging and Alternative Biosensing Approaches

In addition to conventional electrochemical, mechanical, and optical biosensors, alternative transduction strategies have been investigated for GFAP detection, including magnetoimpedance (MI), field-effect transistors (FETs), and terahertz (THz) metasurface-based platforms. These approaches exploit changes in magnetic, electrical, or electromagnetic properties induced by GFAP recognition and offer interesting possibilities for highly sensitive and miniaturized detection.

Sayad et al. (2022) [97] developed a magnetoimpedance (MI) immunosensor specifically designed to differentiate stroke subtypes (IS vs. ICH) using whole-blood and plasma samples. The platform combines a microfluidic chip for GFAP extraction from small-volume blood samples with an MI biosensor. GFAP molecules are first captured by antibodies conjugated to magnetic Dynabeads and subsequently detected on a gold film functionalized with anti-GFAP antibodies, forming a sandwich immunoassay. The presence of the captured GFAP alters the magnetic response of the sensing system, which is measured through the magnetoimpedance effect. Using recombinant GFAP, the platform demonstrated an LDR of 0.01–1.0 ng/mL. The system was designed to process whole blood and incorporated microfluidic sample handling, making it particularly relevant to decentralized stroke diagnostics. Although the LOD increased from 0.01 ng/mL for recombinant GFAP to 1.0 ng/mL when six clinical whole-blood and six plasma samples were analyzed (GFAP concentrations measured in plasma using ELISA: 150, 220, 590, 1 × 10^3^, 4.5 × 10^3^ and 9.6 × 10^3^ pg/mL), they were still able to distinguish ICH from AIS, as the cutoff value is in concordance with the LOD, according to the authors. The immunoassay required more than 85 min to be completed, indicating that further improvements in sensitivity, assay time, and stability are required [97].

**Figure 7:**
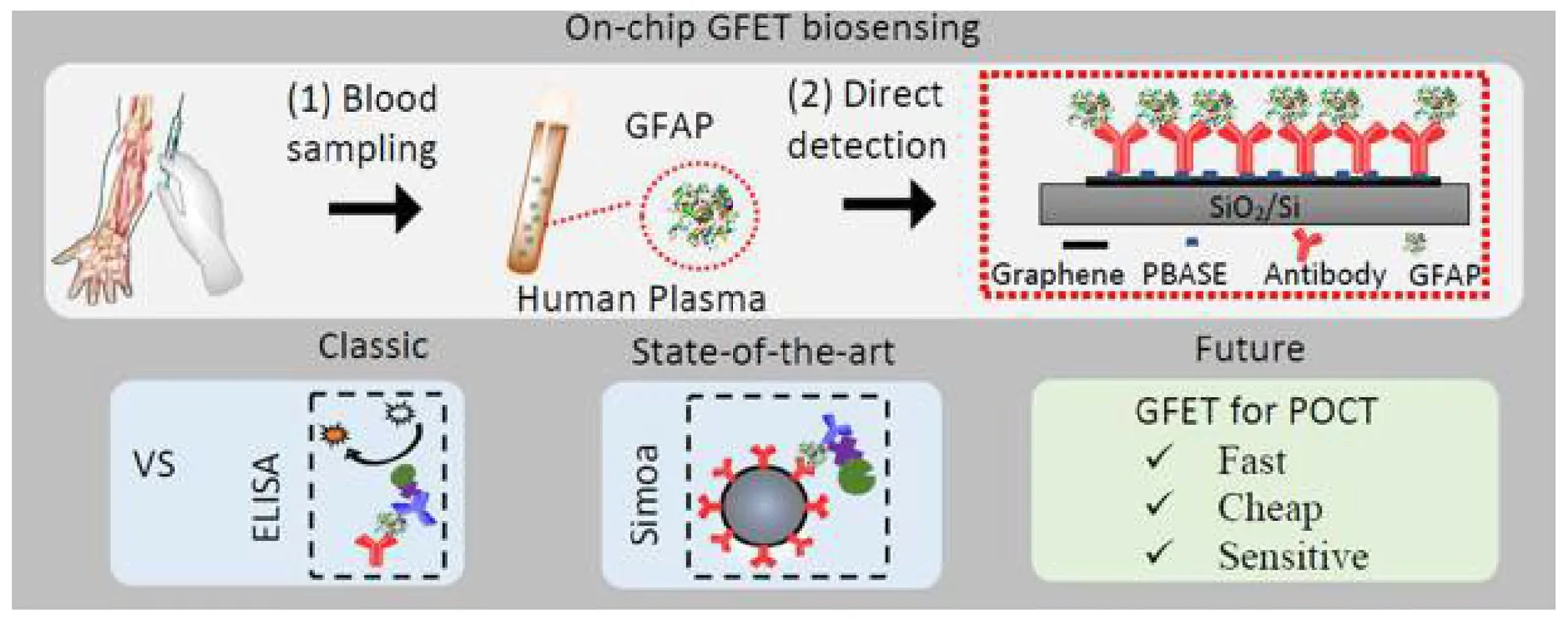
On-chip GFET biosensing platform for GFAP detection using an anti-GFAP-functionalized graphene channel and an integrated reference electrode for liquid gating. GFAP detection is based on the shift in the Dirac point induced by antigen binding. Reproduced from Xu et al. (2021) [98] under the Creative Commons Attribution 4.0 International (CC BY 4.0) licence.

Xu et al. (2021) [98] developed a graphene field-effect transistor (GFET) biosensor for GFAP detection in plasma, with the objective of enabling diagnosis and monitoring of patients with suspected TBI in general practitioner settings and at home (Figure 7). The device consists of a monolayer graphene FET functionalized with specific antibodies against GFAP. When GFAP binds to the antibody-functionalized graphene surface, the electrical properties of the transistor change, resulting in a shift in the Dirac point that correlates with the GFAP concentration. The sensor achieved an LOD of 231 fg/mL using seven samples of patient plasma, a sensitivity of 0.0056 V/(pg/mL), and an LDR from 231 fg/mL to 230 pg/mL. The platform also demonstrated high specificity and selectivity and a sample-to-result time below 15 min. These characteristics, combined with the potential for portable and low-cost implementation, make the GFET approach particularly interesting for decentralized GFAP detection. However, plasma viscosity can affect the effective concentration range, while plasma preparation remains necessary before analysis [98].

More recently, Liang et al. (2026) [99] developed a microfluidic-integrated quasi-bound state in the continuum (Q-BIC) THz metasurface biosensor for the detection of GFAP antigens and antibodies to monitor glioma. The platform consists of an asymmetric aluminum split-ring resonator array fabricated on a quartz substrate and integrated with an SU-8 microfluidic channel. The structural asymmetry generates Q-BIC resonances with a highly confined electromagnetic field, enhancing the interaction between the metasurface and target molecules. Binding of GFAP changes the local dielectric environment surrounding the resonator, resulting in concentration-dependent changes in the THz transmission spectrum. These changes were measured using THz time-domain spectroscopy, with continuous wavelet transform and differential analysis used to improve the detection of weak spectral responses. The sensor achieved LODs of 0.77 pg/mL for GFAP antigens and 1.03 pg/mL for GFAP antibodies, with an LDR of 1 pg/mL–100 ng/mL, using GFAP standard solutions diluted in PBS. Despite the promising analytical performance and microfluidic integration, the experiments were primarily conducted in PBS, meaning that specificity and selectivity in complex clinical matrices remain to be established. Furthermore, the use of a THz time-domain spectroscopy system currently requires top bench equipment, which limits portability, while sample-to-result time and long-term microfluidic stability have not yet been established [99].

Overall, these alternative approaches demonstrate the potential of non-conventional transduction mechanisms for highly sensitive GFAP detection. The studies also illustrate different strategies for moving towards decentralized testing, ranging from whole-blood processing and microfluidic integration to portable transistor-based detection. However, their current clinical applicability remains limited by factors such as sample processing, assay duration, the use of simplified matrices, and the need for further miniaturization and validation.

#### 4.1.6. Comparative Analysis of GFAP Biosensors

The different biosensor platforms investigated for GFAP detection demonstrate considerable variation in transduction mechanisms, analytical performance, biological matrices, assay duration, and degree of integration [81,82,83,84,85,86,87,88,89,90,91,92,93,94,95,96,97,98,99]. Importantly, the studies also provide different levels of evidence regarding their potential applicability to stroke. Based on their intended application, the studies can broadly be classified into three groups: stroke-related applications, applications in other neurological conditions, and general biosensor development where no specific clinical application was stated. However, this classification should be considered together with the type of experimental evidence provided. In particular, the studies differ according to whether GFAP detection was demonstrated using clinical samples from stroke patients, clinical samples from neurological conditions other than stroke, spiked biological matrices, or only standard solutions and other analytical matrices. Therefore, an intended application related to stroke does not necessarily imply clinical validation in stroke patients. Similarly, potential transferability to stroke cannot be considered evidence of stroke diagnostic performance, particularly when the sensor has only been evaluated in other neurological conditions, artificial or spiked matrices, or under controlled analytical conditions.

Among the studies specifically targeting stroke, Rioboó-Legaspi et al. (2026) [87] demonstrated GFAP detection using four clinical serum samples from stroke patients (280, 1.6 × 10^3^, 4.74 × 10^3^ and 12.2 × 10^3^ pg/mL), which were further diluted (1:10 in 10 mM PBS 7.4) before analysis using the eVFIA [87]. Sayad et al. (2022) [97] evaluated their immunoassay using six clinical whole-blood and six clinical plasma samples (150–9.6 × 10^3^ pg/mL). Although the LOD increased from 0.01 ng/mL for recombinant GFAP to 1.0 ng/mL when analyzing the clinical samples, the platform was able to distinguish ICH from AIS, as the cutoff value is in concordance with the LOD, according to the authors [97]. Therefore, both studies provided direct clinical evidence supporting the applicability of their platforms to stroke, although this evidence was limited by the small number of clinical samples analyzed [87,97]. Wang et al. (2025) [95] provided a larger clinical dataset, analyzing fifty serum samples from stroke patients to distinguish HS (*n* = 25) from IS (*n* = 25). The sensor achieved a cutoff value of 2.07 ng/mL, with an AUC of 0.998, demonstrating strong discriminatory performance between the two stroke subtypes. Since the study used clinical samples from stroke patients, these results provide direct evidence of the diagnostic potential of the platform. However, the relatively small cohort and the absence of independent external validation limit the generalisability of the reported performance. Therefore, these findings should be considered promising evidence of diagnostic potential rather than definitive evidence of established clinical utility [95]. In contrast, Kakkar et al. (2025) [90] did not report validation using clinical human samples. Although this study demonstrated analytical feasibility, with reported LOD and LDR values compatible with GFAP concentrations expected in stroke, its performance was established using spiked biological matrices (*n* = 8: 5–5 × 10^3^ pg/mL) rather than clinical samples from stroke patients and therefore cannot be considered clinical validation. This distinction is particularly relevant when interpreting the analytical ranges of these platforms [90].

For biosensors developed for other neurological conditions or for general GFAP detection, the analytical range is particularly relevant when assessing their potential applicability to stroke. Several platforms reported LDRs and LODs that are potentially compatible with GFAP concentrations relevant to biological matrices observed in stroke, including the systems developed by Li et al. (2024) [81], Özcelikay et al. (2022) [82], Salahandish et al. (2022) [84], Xiao et al. (2025) [91], Han et al. (2025) [92], Indongo et al. (2025) [94], Cao et al. (2026) [96], and Liang et al. (2026) [99]. Reported GFAP concentrations during the early hyperacute phase varied considerably between studies, reflecting differences in patient populations, biological matrices, disease subtype, and analytical methods [35,36,61,64,65,66]. Therefore, compatibility between a biosensor’s analytical range and the reported clinical concentrations was considered in relation to the extent of overlap with concentrations reported across these studies. Platforms whose LODs and LDRs overlap with GFAP concentrations reported in one or more early hyperacute stroke studies may have potential applicability to early stroke assessment, whereas platforms covering concentrations reported across a broader range of studies provide stronger analytical compatibility. However, such overlap should not be interpreted as evidence of clinical suitability, as diagnostic performance can only be established through validation using appropriately sized clinical cohorts collected within the intended pre-hospital or early hospital time window.

Among these, Li et al. (2024) [81] demonstrated the performance of their platform using three real plasma samples, while Cao et al. (2026) [96] analyzed twelve real serum samples. Although these studies demonstrate that the biosensors can operate in clinically derived biological matrices, the samples were not obtained from patients with stroke, were limited to small sample numbers, and therefore do not constitute clinical validation for stroke. Rather, they provide evidence of compatibility with real biological samples [81,96]. Salahandish et al. (2022) [84] used GFAP-spiked plasma (*n* = 3: 50, 100, and 500 pg/mL), Han et al. (2025) [92] used GFAP-spiked serum (*n* = 4: 510, 890, 1.37 × 10^3^ and 1.86 × 10^3^ pg/mL), and Indongo et al. (2025) [94] evaluated GFAP-spiked serum, urine, and saliva, but only qualitatively. These studies demonstrate analytical feasibility in biological matrices but do not provide evidence of performance in clinical samples [84,92,94]. Lastly, Özcelikay et al. (2022) [82], Xiao et al. (2025) [91], and Liang et al. (2026) [99] reported compatible analytical ranges based on controlled experimental conditions but did not evaluate their platforms using clinical human samples.

In contrast, Xu et al. (2021) [98] and Zheng et al. (2022) [93] reported LODs and LDRs that do not overlap with the GFAP concentrations expected in stroke cases but could potentially be adapted through dilution of the clinical sample. Özcan et al. (2025) [89] reported an analytical range substantially below the GFAP concentrations relevant to its intended application in TBI diagnosis and therefore used a 10^5^-fold dilution. For stroke, an even greater dilution would be required, as GFAP concentrations typically observed in serum following stroke are higher [35,64,65,89].

Importantly, evaluation using clinical samples does not by itself establish diagnostic utility. The reported diagnostic performance should be interpreted in relation to the number of clinical samples analyzed, the statistical power of the study, the confidence intervals of the performance estimates, and whether the results were confirmed in an independent cohort. Studies based on small clinical cohorts may provide preliminary evidence of diagnostic potential, but their results may not be representative of the broader stroke population. Therefore, larger and adequately powered clinical studies, preferably including independent external validation, are necessary to determine whether the analytical performance of these biosensors can translate into reliable and generalisable diagnostic performance.

The biological matrix and analysis time also strongly influence the potential for early hyperacute stroke diagnosis. Non-invasive fluids, such as urine, sweat, and saliva, have not yet been sufficiently characterized for GFAP detection in stroke cases [62,68,69]. Therefore, platforms such as those developed by Shahub et al. (2022) [83], Mehmandoust et al. (2022) [86], and Indongo et al. (2025) [94] could potentially be investigated as non-invasive alternatives for GFAP detection in stroke [83,86,94]. As previously discussed, serum and plasma are commonly used as clinically relevant matrices, but require additional processing, whereas whole-blood analysis, such as that demonstrated by Sayad et al. (2022) [97], could simplify the workflow [61,97]. However, this platform requires a relatively long assay time, limiting its potential usability in pre-hospital care [97]. Several platforms reported relatively short detection times, including Salahandish et al. (2022) [84], Li et al. (2024) [81], Rioboó-Legaspi et al. (2026) [87], Wang et al. (2025) [95], and Xu et al. (2021) [98]. However, the reported assay time generally reflects the interval from sample addition to result readout and does not necessarily account for sample preparation and processing steps, such as dilution, centrifugation, filtration, incubation, and washing. Furthermore, the total sample-to-answer time was not consistently reported across studies, limiting direct comparisons of their practical turnaround times.

Regarding the recognition element, antibodies were the predominant choice across the reviewed GFAP biosensors, reflecting their high specificity and established use in biosensors [82,83,84,85,86,87,88,89,90,91,92,93,94,95,97,98]. However, alternative recognition strategies were also explored. Li et al. (2024) [81] employed an MIP as a synthetic recognition element, providing an antibody-free approach for selective GFAP recognition [81]. Cao et al. (2026) [96] further demonstrated the potential of GFAP-specific DNA aptamers as an alternative biorecognition element, showing good selectivity and specificity, although the platform remains at the proof-of-concept stage [96]. In addition, Liang et al. (2026) [99] demonstrated a different approach based on direct interaction of GFAP antigens and antibodies with a Q-BIC THz metasurface, without relying on a conventional molecular recognition element [99]. Overall, these approaches highlight the potential of alternative recognition strategies to complement conventional antibodies and broaden the design possibilities for GFAP biosensors [81,96,99].

In terms of portability and clinical translation, electrochemical and FET-based platforms generally show strong potential for miniaturization and integration with portable readout systems, while some optical approaches still rely on relatively specialized instrumentation [81,82,83,84,85,86,87,90,91,92,93,94,96,98]. For a biosensor to be suitable for PoC use, additional practical factors must be considered, including the need for sample preparation, whole-blood compatibility, reagent stability during storage and operation, temperature stability, calibration requirements, user intervention, environmental conditions, and energy consumption. Sample preparation steps such as filtration, centrifugation, dilution, or the addition of reagents may substantially increase workflow complexity and limit the practical portability of otherwise miniaturized platforms. Similarly, microfluidic integration does not necessarily imply compatibility with whole blood, which may introduce challenges related to viscosity, cellular components, and channel performance. Therefore, true POC readiness requires not only a miniaturized sensing and readout system, but also an integrated workflow capable of producing reliable results with minimal sample handling, stable reagents, simple calibration and operation, and limited dependence on external equipment or power sources.

Based on these criteria, none of the reviewed biosensors can yet be considered fully portable, as the complete diagnostic workflow remains limited by sample preparation requirements, external instrumentation, or insufficient integration. The wearable electrochemical biosensor developed by Shahub et al. [83] offers promising portability features through its wearable format, portable readout, and wireless communication. However, GFAP detection was demonstrated using a spiked sweat analogue, and the clinical relevance of sweat GFAP for early-hyperacute stroke assessment remains to be established, requiring validation with real sweat [83]. Another promising approach is the magnetoimpedance biosensor developed by Sayad et al. [97], which incorporates microfluidic processing and enables GFAP detection in whole blood, potentially reducing dependence on conventional blood separation procedures. Nevertheless, its assay time of more than 85 min and the need for further improvements in analytical performance and operational robustness limit its current suitability for rapid PoC testing [97].

Beyond the analytical performance and portability of individual GFAP biosensors, multiplexed platforms may provide additional clinical value by enabling the simultaneous detection of complementary biomarkers, as stated before. Shahub et al. (2022) [83], for example, demonstrated the simultaneous detection of GFAP and the inflammatory biomarker IL-6 in sweat using a wearable platform for continuous monitoring of TBI [83]. More directly relevant to stroke, Wang et al. (2025) [95] developed a multiplexed SERS immunosensor for the simultaneous detection of GFAP and RBP4 in serum, demonstrating its application to the discrimination of IS and HS in clinical samples. However, because the diagnostic performance of the biomarkers was assessed separately, the study does not establish whether combining GFAP and RBP4 improves diagnostic discrimination compared with either marker alone [95]. These findings suggest that multiplexed biosensing could extend the clinical utility of GFAP detection by combining information related to neurological injury with complementary pathological processes and, in the case of stroke, potentially supporting subtype differentiation.

Overall, the most promising platforms for early hyperacute stroke are those that combine an analytical range compatible with clinically relevant GFAP concentrations with rapid analysis, microfluidic platforms requiring minimal sample preparation, portable instrumentation and the capability of multiplexed detection. The key characteristics of the biosensors discussed throughout this section, including their transduction mechanisms, recognition element, biological matrices, analytical ranges, detection limits, sensitivity, assay times, and clinical validation, are summarized in Table 3. Nevertheless, no platform currently demonstrates all these characteristics together with extensive clinical validation, highlighting the need for further development before GFAP biosensors can be routinely implemented in decentralized stroke diagnosis.

## 5. Conclusions and Future Perspectives

Rapid and accurate stroke diagnosis remains a major clinical challenge, particularly during the early hyperacute phase, when treatment decisions are highly time-dependent. This review highlights the potential of blood-based biomarkers and biosensing technologies to complement conventional diagnostic approaches and support faster PoC assessment. Among the biomarkers evaluated, GFAP represents a promising candidate for early hyperacute stroke assessment, particularly for differentiating ICH from IS and SMs. Its reported increase following haemorrhagic injury, together with its reported diagnostic and prognostic value, makes GFAP an attractive biomarker for investigation in pre-hospital and PoC applications.

The reviewed biosensor platforms demonstrate that GFAP can be detected using a variety of transduction strategies, including electrochemical, optical, and mechanical approaches. Several systems combine high analytical sensitivity with miniaturization, reduced assay times, and potential compatibility with portable or integrated platforms. However, differences in biological matrices, sampling times, analytical methods, patient populations, and reported diagnostic thresholds are still not fully explored, and currently limit direct comparison between studies, hindering the establishment of standardized performance criteria. Furthermore, most reported biosensors remain at the analytical or early validation stage and require broader clinical evaluation. Results obtained from small clinical cohorts should be interpreted cautiously, as high discriminatory performance in limited datasets does not necessarily translate into generalizable diagnostic utility. Larger, adequately powered studies with appropriate statistical evaluation and independent external validation are therefore needed to establish the robustness and clinical applicability of these platforms.

Future research should therefore focus on clinically validated, robust, and user-friendly platforms capable of rapid GFAP detection in minimally processed blood samples. In this context, advanced microfluidic technologies offer an important route toward the development of automated sample-to-answer PoC systems [100,101,102]. Microfluidic sample-processing strategies can integrate operations such as plasma separation and filtration, and other sample-preparation steps while reducing processing time and manual intervention [100,101]. Integrating these sample-processing steps before the sensing stage could simplify the assay workflow and may facilitate automated GFAP analysis from blood samples [100]. Furthermore, the development of low-cost polymeric and paper-based microfluidic architectures could improve the accessibility and portability of these systems [101,103]. These platforms enable precise handling and mixing of small volumes of samples and reagents, reducing reagent consumption while allowing controlled fluid transport and reaction conditions [101,103]. Moreover, direct immobilization of biorecognition elements within microfluidic channels or on integrated sensing surfaces could further integrate sample processing, target capture, and signal transduction within a single device [101,104]. Appropriate surface-functionalization strategies are essential to ensure the stability and performance of the immobilized recognition elements, which can directly affect assay sensitivity, specificity, and reproducibility [102,105]. Such integration could ultimately support the development of compact and automated microfluidic platforms in which sample preparation, controlled mixing, biomolecular recognition, and signal detection are performed within a single device [101].

Further optimization of the biorecognition element may also improve biosensor specificity, stability, and robustness. Alternative recognition strategies to conventional antibodies, such as aptamers and MIPs, could represent promising approaches for future GFAP sensing platforms [81,96,105]. Multiplexed biosensors combining GFAP with complementary biomarkers may provide additional diagnostic and prognostic information and could potentially improve stroke classification and patient stratification [95,106]. Furthermore, combining GFAP with complementary biomarkers and applying machine learning algorithms to multiplexed biosensor data may offer opportunities to improve stroke diagnosis, subtype classification, and patient stratification [107,108]. By integrating information from distinct pathophysiological processes, such approaches may help address diagnostic uncertainty when GFAP alone is insufficient to distinguish ICH, IS, and stroke mimics. Nevertheless, the incremental diagnostic value of these combinations remains to be established through careful and adequately powered clinical studies that include representative populations of patients with ICH, IS, and stroke mimics. Ultimately, the integration of GFAP biosensing with portable, low-cost, and low-power PoC technologies, including automated sample-processing microfluidic devices, represents a promising direction for future research. However, substantial clinical validation is still required before such systems can be considered reliable tools for routine stroke diagnosis or as alternatives to established clinical assessment and imaging.

## Figures and Tables

**Table 1:** Summary of main stroke biomarkers.

Biomarker Class	Biomarker	References	Biological Role/Source	Temporal Kinetics	Diagnostic Utility	Prognostic Value	Pre-Hospital/PoC Suitability
Structural and Neural Injury	GFAP	[25,30,32,37,38,39,40,41,42,43]	Intermediate filament protein in astrocytes	Detectable within minutes; peaks in 2–6 h (ICH)	High specificity for ICH; distinguishes ICH vs. IS and SMs	Predicts infarct volume, severity, haemorrhagic transformation, and reperfusion safety	High
	NfL	[37,38,44]	Neuron-specific cytoskeletal protein	Rises days after onset; peaks in 14–21 days	Limited early hyperacute diagnostic value	Robust marker of neuroaxonal damage	Low
	NSE	[37,38]	Glycolytic isoenzyme in neurons and neuroendocrine cells	Rises in 3–12 h	Marker of neuronal damage but insufficient alone for early diagnosis	Limited early prognostic use	Low/Moderate
	S100B	[25,38,41]	Calcium-binding protein mainly in astrocytes	Rises in 3–12 h; peaks in 2–3 days	Limited standalone diagnostic utility	Predicts malignant infarction, cerebral edema, haemorrhagic transformation	Low
BBB Stability and Matrix Degradation	MMP-9	[25,41,44,45,46]	Zinc-dependent endopeptidase involved in ECM degradation	Early elevation	Poor discrimination of IS vs. HS	Strong predictor of haemorrhagic transformation, edema, infarct size, reperfusion safety	High
	Occludin	[25,30,45]	Tight junction protein of BBB	Released rapidly; high within the first 12 h	Elevated in acute IS vs. SMs	Reflects BBB disruption	Moderate
	Claudin-5	[25,30,45]	Tight junction protein of BBB	Released rapidly; high within the first 12 h	Strong indicator of vascular permeability and IS	Reflects early BBB compromise	Moderate
	ZO-1	[25,30]	Tight junction scaffolding protein	Early elevation	Supports stroke subtype differentiation	Indicates BBB structural disruption	Moderate
Inflammation and Oxidative Stress	IL-6	[25,37,38,41,44]	Neuroinflammatory cytokine	May double within ~26 min; prognostic window, 24–72 h	Good sensitivity but low specificity; limited standalone use	Prognostic during acute inflammatory response	Low
	TNF-α	[25,37,38,41,44]	Pro-inflammatory cytokine from microglia/macrophages	Released within ~4 h	Moderate specificity, low sensitivity	Predicts infarct size, inflammation, infection risk, poor outcomes	Low
	CRP	[37,38]	Systemic inflammatory protein	Subacute elevation	Poor acute diagnostic utility	Strong prognostic indicator in IS	Low
Coagulation and Fibrinolysis	D-dimer	[25,37,38,44]	Fibrin degradation product	Elevated within minutes; peaks in 3–24 h	Low specificity and sensitivity for stroke subtype diagnosis	Predicts clot burden, haemorrhagic transformation, recurrent events	Moderate/High
Cardiac and Systemic Stress	TnI	[25,37,38,41,47,48,49,50,51,52,53]	Cardiac troponin associated with myocardial injury	Rapid early elevation	Useful for excluding SMs and identifying cardiac involvement	Predicts severity, mortality, and functional outcome	Moderate
	BNP/NT-proBNP	[25,37,41,54,55,56]	Cardiac stress peptides	Elevated early	Limited mimic discrimination; NT-proBNP higher in HS and cardioembolic stroke	Predicts cardioembolic etiology, severity, and mortality	Moderate

**Table 2:** Summary of studies investigating GFAP concentrations in blood-based matrices in patients with acute stroke.

Fluid	Authors	Method	Concentration in SMs ^1^	Concentration in IS ^1^	Concentration in HS ^1^	Cutoff Point (for ICH)
Whole Blood	Xue et al. [61]	Chemiluminescence immunoassay in acute phase of stroke	-	*n* = 52; 78.84 pg/mL [37.22–145.30]	*n* = 27; 549.95 pg/mL [171.40–4239]	-
Serum	Jæger et al. [64]	Measured within 4.5 h of symptom onset using Simoa^®^	*n* = 114; 192 pg/mL [109–386]	*n* = 131; 268 pg/mL [171–514]	ICH: *n* = 21; 1484 pg/mL [310–5370]; SAH: *n* = 4; 718 pg/mL [381–12,496]	5369 pg/mL: 25% sensitivity (95% CI: 11.5–43.4) and 100% specificity (95% CI: 98.6–100.0)
	Luger et al. [35]	Measured within 6 h of symptom onset using an electrochemiluminometric immunoassay	*n* = 157; 10 pg/mL [10–10]		ICH: *n* = 45; 160 pg/mL [40–3270]	30 pg/mL (AUC = 0.872; 95% CI: 0.802–0.942; *p* < 0.001)
	Ren et al. [65]	Measured within 4.5 h of symptom onset using ELISA	-	*n* = 79; 20 pg/mL [4–80]	ICH: *n* = 45; 810 pg/mL [180–3310]; SAH: *n* = 5; 1330 pg/mL [20–3210]	340 pg/mL (AUC = 0.86; 95% CI: 0.73–0.99)
Plasma	Kalra et al. [66]	Measured within 6 h of symptom onset with i-STAT-Alinity	*n* = 19; 48 pg/mL [29–97]	*n* = 258; 30 pg/mL [29–51]	ICH: *n* = 76; 208 pg/mL [60–5907]	Dependent on age; general: 55 pg/mL (AUC = 0.880; 95% CI: 0.834–0.925; *p* < 0.001)
	Xue et al. [61]	Chemiluminescence immunoassay in acute phase of stroke	-	*n* = 52; 84.18 pg/mL [49.64–147.25]	*n* = 27; 582.4 pg/mL [182.83–5219.75]	-
	Han et al. [67]	Measured within 6 h of symptom onset using Simoa^®^	-	*n* = 51; 213.8 pg/mL [161.8–439.4]	*n* = 22; 2171.0 pg/mL [922.8–5883]	703 pg/mL (AUC = 0.803; 95% CI: 0.732–0.863)
	Katsanos et al. [36]	Measured within 6 h of symptom onset using chemiluminescent immunoassay	*n* = 31; 70 pg/mL [0–240]	*n* = 121; 110 pg/mL [0–270]	ICH: *n* = 34; 2170 pg/mL [550–10,120]; SAH: *n* = 5; 0 pg/mL [0–220]	430 pg/mL: 91% sensitivity (95% CI: 77–98) and 97% specificity (95% CI: 93–99)

^1^ The concentrations are expressed as (number of samples; median [IQR]). A hyphen (-) indicates that the study did not report any values for the specific condition listed in that column.

**Table 3:** Summary of developed GFAP biosensors (when no data is reported by the authors of the study, the space is left blank).

Application		Reference	Detection Method	Recognition Element	Samples	Performance (LOD/LDR/Sensitivity)	Assay Time ^1^	Clinical Samples	Potential Stroke Applicability
Stroke	Stroke assessment	Rioboó-Legaspi et al. (2026) [87]	Electrochemical Chronoamperometric Immunosensor	Antibodies	Serum	85 pg/mL (blocking solution)	<10 min	Diluted Serum (real sample) (*n* = 4: 280–12.2 × 10^3^ pg/mL)	LOD/LDR (standard solution) overlap with GFAP concentrations reported in some early-hyperacute stroke studies. Partially portable. Demonstrated with a limited cohort of clinical samples from stroke patients.
						100–10 × 10^3^ pg/mL (blocking solution)			
						-			
	Stroke and SM differentiation	Kakkar et al. (2025) [90]	Optical UCNP Immunosensor	Antibodies	Serum	82 × 10^−3^ pg/mL (20% serum)	>2 h	Diluted serum spiked with GFAP (*n* = 8: 5–5 × 10^3^ pg/mL)	LOD/LDR (spiked serum) overlap with GFAP concentrations reported in some early-hyperacute stroke studies. Not demonstrated as portable. Demonstrated only with spiked serum.
						10–5 × 10^3^ pg/mL (20% serum)			
						-			
	Stroke subtype differentiation	Wang et al. (2025) [95]	Optical SERS Immunosensor	Antibodies	Serum	0.16 pg/mL (standard solution)	~30 min	Serum (real sample) (*n* = 50)	LOD/LDR (standard solution) overlap with GFAP concentrations reported in early-hyperacute stroke studies. Not demonstrated as portable. Demonstrated with a cohort of clinical samples from stroke patients.
						0.1–0.1 × 10^6^ pg/mL (standard solution)			
						-			
	Stroke subtype differentiation	Sayad et al. (2022) [97]	Magnetoimpedance Immunosensor	Antibodies	Blood	10 pg/mL (PBS) 10^3^ pg/mL (blood and plasma)	<30 min	Plasma (*n* = 6) and blood (*n* = 6) (real samples) (150–9.6 × 10^3^ pg/mL)	LOD/LDR (standard solution) overlap with GFAP concentrations reported in some early-hyperacute stroke studies. Partially portable. Demonstrated with a limited cohort of clinical samples from stroke patients.
						10–10^3^ pg/mL (PBS)			
						-			
Neurological Conditions	Neurological disease assessment	Li et al. (2024) [81]	Electrochemical rGO/PDA-MIP-Based DPV Biosensor	MIP	Plasma or CSF	754.5 × 10^−6^ pg/mL (PBS)	~10 min	Plasma (real sample) (*n* = 3: 37–56,424 pg/mL)	LOD/LDR (standard solution) overlap with GFAP concentrations reported in early-hyperacute stroke studies. Not demonstrated as portable. Demonstrated with a limited cohort of clinical samples.
						10^−3^–10^3^ pg/mL (PBS)			
						2.13 µA/log(ag/mL) (PBS)			
	Central nervous system injury monitoring	Özcelikay et al. (2022) [82]	Electrochemical Impedance Immunosensor	Antibodies	Serum	51 × 10^−3^ pg/mL (standard solution)	~1 h	-	LOD/LDR (standard solution) overlap with GFAP concentrations reported in some early-hyperacute stroke studies. Not demonstrated as portable. Not validated with clinical samples.
						1–10^3^ pg/mL (standard solution)			
						0.308 Ω/(pg/mL) (synthetic serum)			
	TBI continuous prognostic monitoring	Shahub et al. (2022) [83]	Electrochemical Impedance Immunosensor	Antibodies	Sweat	14 pg/mL (sweat analogue)	-	-	Sweat GFAP not established in stroke. Partially portable. Not validated with clinical samples.
						-			
						-			
	TBI, spinal cord injury, and brain stroke detection	Salahandish et al. (2022) [84]	Electrochemical Impedance Immunosensor	Antibodies	Plasma	3 pg/mL (PBS)	<30 min	Plasma spiked with GFAP (*n* = 3: 50, 100 and 500 pg/mL)	LOD/LDR (standard solution) overlap with GFAP concentrations reported in some early-hyperacute stroke studies. Partially portable. Demonstrated only with spiked plasma.
						10–10^3^ pg/mL (PBS)			
						39 mL/(pg mm^2^) (PBS)			
	TBI diagnosis	Mehmandoust et al. (2022) [86]	Electrochemical Impedimetric Immunosensor	Antibodies	Urine	0.05 pg/mL (PBS)	>45 min	Diluted urine spiked with GFAP (*n* = 3: 0.1, 0.2, 3.5 pg/mL)	Urine GFAP not established in stroke. Not demonstrated as portable. Demonstrated only with spiked urine.
						0.05–10 pg/mL (PBS)			
						-			
	Multiple brain-pathology detection	Agostini et al. (2021) [88]	Mechanical SAW Immunosensor	Antibodies	Serum	35 pM (PBS and serum-matrix)	-	-	LDR not established. Not demonstrated as portable. Not validated with clinical samples.
						-			
						-			
	TBI diagnosis	Özcan et al. (2025) [89]	Mechanical Mass-Sensitive QTF Immunosensor	Antibodies	Serum	-	>30 min	Commercial serum spiked with GFAP (*n* = 6)	LDR does not adequately overlap reported early-hyperacute concentrations (dilution required). Not demonstrated as portable. Demonstrated only with spiked serum.
						50 × 10^−6^–25 × 10^−3^ pg/mL (PB)			
						0.2729 Hz/(fg/mL) (PB)			
	TBI diagnosis	Xiao et al. (2025) [91]	Optical Microfiber Interference Immunosensor	Antibodies	Serum and CSF	0.21 aM (10% fetal bovine serum) 0.10 aM in (10% artificial CSF) 0.09 aM (PBS)	-	-	LOD/LDR (standard solution) overlap with GFAP concentrations reported in some early-hyperacute stroke studies for serum, CSF not studied in early hyperacute phase of stroke. Not demonstrated as portable. Not validated with clinical samples.
						1 aM–0.1 nM (PBS)			
						0.324 nm/log(M) (10% artificial CSF) 0.305 nm/log(M) (10% fetal bovine serum)			
	Tumour (gliomas) diagnosis	Han et al. (2025) [92]	Optical ECL Immunosensor	Antibodies	Serum	1.66 pg/mL (PBS)	>2 h	Serum spiked with GFAP (*n* = 4: 510–1.86 × 10^3^ pg/mL)	LOD/LDR (standard solution) overlap with GFAP concentrations reported in early-hyperacute stroke studies. Not demonstrated as portable. Demonstrated only with spiked serum.
						5–100 × 10^3^ pg/mL (DR) (PBS)			
						812.59 a.u./log(ng/mL) (PBS)			
	TBI diagnosis	Zheng et al. (2022) [93]	Optical NIR Plasmonic Fluorescent Immunosensor	Antibodies	Plasma	0.6 pg/mL (commercial plasma)	>1 h	Commercial plasma spiked with GFAP (*n* = 13: 1.0–100 × 10^3^ pg/mL)	LDR does not adequately overlap reported early-hyperacute concentrations (dilution required). Partially portable. Demonstrated only with spiked commercial plasma.
						1–500 pg/mL (commercial plasma)			
						127 a.u./log(pg/mL) (commercial plasma)			
	Tumour (gliomas) diagnosis	Indongo et al. (2025) [94]	Optical FRET Fluorescent Immunosensor	Antibodies	Serum, urine and saliva	32 pg/mL (probe solution)	Real time	Serum, urine and saliva spiked with GFAP	LOD/LDR (standard solution) overlap with GFAP concentrations reported in early-hyperacute stroke studies for serum, saliva and urine not studied in hyperacute phase. Partially portable. Demonstrated only with spiked serum, urine and saliva.
						0–46 × 10^3^ pg/mL (probe solution)			
						-			
	Alzheimer’s early diagnosis	Cao et al. (2026) [96]	Optical Fluorescent Biosensor	Aptamers	Serum	0.24 pg/mL (spiked serum)	-	Serum (real sample) (*n* = 12: 88–358 pg/mL)	LOD/LDR (standard solution) overlap with GFAP concentrations reported in early-hyperacute stroke studies. Not demonstrated as portable. Demonstrated with a limited cohort of clinical samples.
						10–10^6^ pg/mL (spiked serum)			
						-			
	TBI diagnosis and monitoring	Xu et al. (2021) [98]	GFET Immunosensor	Antibodies	Plasma	20 × 10^−3^ pg/mL (PBS) 231 × 10^−3^ pg/mL (plasma)	<15 min	Plasma (real sample) (*n* = 7: 231 × 10^−3^–230 pg/mL)	LDR does not adequately overlap with reported early-hyperacute concentrations (dilution required). Partially portable. Demonstrated with a limited cohort of clinical samples.
						231 × 10^−3^–230 pg/mL (plasma)			
						0.0056 V/(pg/mL) (plasma)			
	Tumour (gliomas) diagnosis	Liang et al. (2026) [99]	Electromagnetic Q-BIC THz Metasurface	-	Serum	0.77 pg/mL (GFAP antigens in PBS) 1.03 pg/mL (GFAP antibodies in PBS)	-	-	LOD/LDR (standard solution) overlap with GFAP concentrations reported in early-hyperacute stroke studies. Not demonstrated as portable. Not validated clinically.
						1–100 × 10^3^ pg/mL (PBS)			
						-			
Biosensor Development	GFAP determination in human CSF	García-Rodrigo et al. (2024) [85]	Electrochemical Amperometric Immunosensor	Antibodies	CSF	160 pg/mL (PBS)	~2 h 30 min	CSF (real sample) (*n* = 3: 6 × 10^3^, 27 × 10^3^ and 38 × 10^3^ pg/mL)	CSF GFAP not studied in hyperacute phase of stroke. Not demonstrated as portable. Demonstrated with a limited cohort of clinical samples.
						600–100 × 10^3^ pg/mL (PBS)			
						-			

^1^ Assay time refers to the time elapsed from sample addition to result readout, as reported in the original studies. Portability was classified according to the practicality of the complete diagnostic workflow, including sample preparation, instrumentation, power requirements, and user intervention: portable indicates a demonstrated integrated workflow suitable for decentralized testing; partially portable indicates that portable or miniaturized components are available but relevant workflow limitations remain; and not portable indicates that portability of the complete workflow has not been demonstrated, typically due to reliance on laboratory equipment or substantial sample processing.

## Data Availability

No new data were created or analyzed in this study. Data sharing is not applicable to this article.

## Abbreviations

The following abbreviations are used in this manuscript:

AUC	Area Under the Curve
BBB	Blood–Brain Barrier
BE FAST	Balance, Eyes, Face, Arms, Speech, Time
BNP	B-Type Natriuretic Peptide
BSA	Bovine Serum Albumin
CRP	C-Reactive Protein
CSF	Cerebrospinal Fluid
CT	Computed Tomography
DALYs	Disability-Adjusted Life Years
DNA	Deoxyribonucleic Acid
DPV	Differential Pulse Voltammetry
ECL	Electrochemiluminescence
ECM	Extracellular Matrix
ELISA	Enzyme-Linked Immunosorbent Assay
eVFIA	Electrochemical Vertical-Flow Immunoassay
FAST	Face, Arms, Speech, Time
FET	Field-Effect Transistor
FRET	Förster Resonance Energy Transfer
GFAP	Glial Fibrillary Acidic Protein
GFET	Graphene Field-Effect Transistor
GO	Graphene Oxide
HCT	Haematocrit
HRP	Horseradish Peroxidase
HS	Haemorrhagic Stroke
ICH	Intracerebral Hemorrhage
IL-6	Interleukin-6
IQR	Interquartile Range
IS	Ischaemic Stroke
LDR	Linear Detection Range
LOD	Limit of Detection
MIP	Molecularly Imprinted Polymer
MMP-9	Matrix Metalloproteinase-9
MRI	Magnetic Resonance Imaging
NfL	Neurofilament Light Chain
NIHSS	National Institutes of Health Stroke Scale
NIR	Near-Infrared
NSE	Neuron-Specific Enolase
NT-proBNP	N-Terminal Prohormone of B-Type Natriuretic Peptide
PB	Phosphate Buffer
PBS	Phosphate-Buffered Saline
PDA	Polydopamine
PoC	Point-of-Care
Q-BIC	Quasi-Bound State in the Continuum
QTF	Quartz Tuning Fork
RBP4	Retinol-Binding Protein 4
rGO	Reduced Graphene Oxide
SAH	Subarachnoid Hemorrhage
SAW	Surface Acoustic Wave
SERS	Surface-Enhanced Raman Scattering
Simoa	Single Molecule Array
SMs	Stroke Mimics
TBI	Traumatic Brain Injury
THz	Terahertz
TIA	Transient Ischemic Attack
TnI	Cardiac Troponin I
TNF-α	Tumour Necrosis Factor α
UCNP	Upconversion Nanoparticle
ZO-1	Zonula Occludens-1
